# Adjuvant chemotherapy after radical nephroureterectomy improves the survival outcome of high-risk upper tract urothelial carcinoma patients with cardiovascular comorbidity

**DOI:** 10.1038/s41598-020-74940-x

**Published:** 2020-10-19

**Authors:** Yong Luo, Bingfu Feng, Dechao Wei, Yili Han, Mingchuan Li, Jiahui Zhao, Yunhua Lin, Zhu Hou, Yongguang Jiang

**Affiliations:** Department of Urology, Beijing Anzhen Hospital, Capital Medical University, Anzhenli Street, Chaoyang District, Beijing, 100029 People’s Republic of China

**Keywords:** Chemotherapy, Urological cancer

## Abstract

This prospective randomized comparative trial study aimed to evaluate the therapeutic outcomes of radical nephroureterectomy and adjuvant chemotherapy (ACT) used in combination in high risk upper tract urothelial carcinoma (UTUC) patients with cardiovascular comorbidity. Based on the inclusion criteria of high-risk UTUC in EAU guidelines (updated in 2014), all eligible patients treated in our hospital from January 2014 to March 2018 were included, and cases with late disease, renal dysfunction, severe cardiopulmonary disease or other malignant tumors were excluded. The cases were randomized into two groups based on treatment regimen. Multivariate analyses were performed to analyze the influencing factors of survival outcome in the enrolled patients. The Cox proportional-hazards model and the Kaplan–Meier method were employed to assess progression free survival (PFS), overall survival (OS) and cancer specific survival (CSS). In addition, the potential adverse effects of chemotherapy were actively monitored. A total of 176 high-risk UTUC individuals with cardiovascular comorbidity were enrolled and evaluated in this study. Median follow-up durations were 30 months (range 6–54) in the RNU (n = 82) group and 36 months (range 6–54) in the RNU + ACT (n = 94) group. Multivariable analysis indicated that peri-operative cardiovascular events risk grade was independent prognostic factor for OS. Tumor size was independent prognostic factor for PFS and CSS. BMI and lymphovacular invasion were significant predictors of PFS. Clinical stage, lymph node involvement, and tumor grade were significant predictors of PFS, OS and CSS in these patients. Especially, chemotherapy was helpful in improving PFS [*P* < 0.001, HR = 6.327 (5.115–7.793)], OS [*P* = 0.013, HR = 2.336 (1.956–2.883)] and CSS [*P* = 0.008, HR = 3.073 (2.533–3.738)]. Kaplan–Meier analysis demonstrated that the oncologic outcomes of RNU treated high-risk UTUC patients were improved much significantly by ACT, including PFS [*P* = 0.0033, HR = 3.78 (3.13–4.55)], OS [*P* = 0.0397, HR = 1.39 (1.01–1.75)] and CSS [*P* = 0.0255, HR = 1.26 (1.07–1.45)]. Further analysis of the lymph node positive subgroup showed that the median time of oncologic events was enhanced in RNU + ACT treated individuals in comparison with the RNU group, including PFS (11.4 months vs. 31.9 months, *P* = 0.0018), OS (26.8 months vs. 36.3 months, *P* = 0.0255) and CSS (28.2 months vs. 39.3 months, *P* = 0.0197). In the T3/4 cohort, significantly increased median PFS (13.9 months vs. 36.3 months, *P* = 0.0217), OS (20.6 months vs. 32.2 months, *P* = 0.0183) and CSS (21.9 months vs. 38.4 months, *P* = 0.0226) were obtained in the combination group. Additionally, no severe adverse events (over grade 4) associated with chemotherapy were detected in the RNU + ACT group. In conclusion, ACT after radical surgery has statistically significant therapeutic effects on PFS, OS and CSS in high-risk UTUC patients with cardiovascular comorbidity.

## Introduction

Upper tract urothelial carcinoma (UTUC) represents a commonly encountered cancer, characterized by high morbidity and mortality. Unlike bladder cancer cases, most UTUC patients show much faster progression, with metastases occurring much earlier^1^. It is well-known that radical nephroureterectomy (RNU) is the preferred approach for UTUC treatment. However, recent findings indicate that high-risk or locally advanced UTUC rapidly progresses again after the RNU procedure. Thus, it is an important challenge faced by urologists to identify ways to further improve the overall survival (OS), cancer specific survival (CSS) and progression free survival (PFS) rates, and to suppress the rapid progression of high-risk or locally advanced UTUC.

In recent years, several experts have suggested that neoadjuvant chemotherapy has a survival benefit in UTUC patients post-operation. Kubota et al.^2^ evaluated 234 patients with high-risk UTUC, and found that neoadjuvant therapy (NAC) could significantly improve the pathological downstaging of the primary lesion as well as lymphovascular invasion (LVI), increasing recurrence-free survival (RFS) and CSS; meanwhile, NAC showed no significant effect on OS. The Young Academic Urologists Urothelial Carcinoma Group of the European Association of Urology meta-analyzed articles prior to April 2016 to comparatively evaluate the role of NAC and adjuvant chemotherapy (ACT) in UTUC after RNU treatment. This comprehensive review suggested that NAC appears promising, with favorable pathologic response rates in patients with UTUC^3^.

Although administration of neoadjuvant cisplatin-based chemotherapeutics has multiple advantages over adjuvant therapy, e.g. improved tolerance in renal function, it is very difficult to perform multicenter prospective randomized trials to evaluate the role of NAC in the UTUC treatment process, because applying endoscopic diagnostic biopsy before NAC in renal pelvis or upper ureter carcinoma is extremely challenging.

Therefore, postoperative adjuvant chemotherapy might be a potentially valuable treatment for individuals not pathologically confirmed as UTUC before surgery. However, because of the undetermined potential therapeutic toxicity to the solitary kidney, it remains largely controversial whether chemotherapy could be used as a regular adjuvant strategy after surgery^4^. To date, no consensus exists regarding the optimal adjuvant approach yielding good outcomes in high-risk UTUC patients following RNU. Especially, these high-risk UTUC patients with cardiovascular comorbidity have potential surgical and anesthetic risks. Therefore, the treatment strategy for this special population is usually very conservative, and their survival is usually much worse. More attention should be paid to these patients and giving more effort to improve prognosis should be the important work and challenge for all urologists.

Therefore, the present prospective randomized comparative trial aimed to evaluate the security and benefit of ACT by assessing oncological outcomes and adverse events in localized high-risk subjects who underwent RNU.

## Patients and methods

### Patients

Consecutive individuals with localized high-risk UTUC, who were diagnosed and treated in Beijing Anzhen Hospitalfrom January 2014 to March 2018, were recruited into this open prospective randomized controlled trial. Three experienced surgeons participated in the present study and were responsible for all surgical operations. The follow-up period ended in September 2018. According to a complete randomized schedule designed by the SAS software, all eligible cases administered RNU were randomly assigned to the observation and ACT groups, respectively. Chemotherapy started one month after operation. Additionally, radical nephroureterectomy was defined as laparoscopic resection of the kidney, ureter and partial bladder, as well as lymphadenectomy. Open surgery is an important alternative when patients are with very high risk of CVE or their intra-operative blood loss was large.

We set up four lymph node dissection areas as the zones (1) from the renal artery to the inferior mesenteric artery, (2) from the inferior mesenteric artery to the abdominal aorta forking, (3) from the abdominal aorta forking to the common iliac artery forking, and (4) below the common iliac artery forking. During surgery, the area of lymph node dissection was determined according to tumor location in each patient. ACT treatment included three cycles (three weeks/cycle) of Gemcitabine plus Cisplatin chemotherapy (three-week GC regimen): Gemcitabine (1000–1200 mg/m^2^, Day 1 and Day 8), Cisplatin (70 mg/m^2^, with total volume divided at Days 2/3/4).

All cases were diagnosed by pathological examination of surgically removed samples. Inclusion criteria in this trial were based on EAU guidelines (updated in 2014). A patient meeting any factor in these inclusion criteria was diagnosed with high-risk UTUC: hydronephrosis, tumor size over 1 cm (cases before 2018), tumor size over 2 cm (cases after 2018), high-grade cytology, multifocal disease, and previous radical cystectomy for bladder cancer or variant histology. Exclusion criteria were: previous neoadjuvant chemotherapy, previous cisplatin-based chemotherapy for bladder cancer, ECOG ≥ 3, preoperative eGFR < 60 mL/min, postoperative eGFR < 60 mL/min (one month after surgery), dysfunction of the contralateral kidney, metastasis, other malignant tumors or severe cardiopulmonary disease, and interruption of follow-up.

It is particularly emphasized that most of the enrolled patients had different types and degrees of cardiac and macrovascular comorbidities, including coronary heart disease, arrhythmia, valvular disease, abdominal aortic aneurysm, ascending aortic aneurysm, and cardiac insufficiency. After discussion within the departments of cardiology, cardiac surgery, macrovascular diseases and anesthesiology, we drew up a peri-operative CVE risk assessment scale (Supplementary file 1). All enrolled patients should not reach the very high risk degree of CVE assessment scale.

This study was approved by the Committee on the Ethics of Clinical Experiments of Capital Medical University (Registration No. CMU2010003X) and performed according to the ethics standards of the 1964 Declaration of Helsinki. It was also registered in the Chinese Clinical Trial Registry that participates in the WHO International Clinical Trial Registry Platform, with registration number ChiCTR1900027924 (04/12/2019). Written informed consent was obtained from each study participant.

### Follow-up protocol and study endpoints

The patients were monitored by cystoscopy for intravesical recurrence every 3 months during the first 2 years, every 6 months for the next 3 years, and then annually thereafter. In addition, cranial computed tomography (CT) or magnetic resonance imaging (MRI) was performed to access disease progression and recurrence every 3 months for the first 2 years, every 6 months for the next 3 years, and yearly in subsequent years. Then, bone radioisotope scanning was generally performed every 12 months in follow-up years.

The primary endpoint was PFS (time to radiographic progression, which was indicated by local failure at the surgical site, regional lymph nodes, or distant metastases). Bladder recurrence was not considered in the analysis of the PFS rate. Secondary endpoints included OS (time to death regardless of cause) and CSS (time elapsed from RNU to cancer death).

### Adverse effect evaluation

By phone calls and questionnaires, possible complications were regularly assessed during the cytotoxic chemotherapy for various system functions, including medullary hematopoiesis function, skin and mucosal system functions, endocrine symptoms, digestive function, cardiovascular events and several important organ functions. All chemotherapy related symptoms were assessed based on Common Terminology Criteria for Adverse Events, version 4.0 (National Cancer Institute, National Institutes of Health, Department of Health and Human Services, May 29, 2009): grade 0, no complication; grade 1, mild symptoms with no treatment required; grade 2, moderate symptoms, generally treated by conservative measures; grade 3, severe symptoms requiring active intervention; grade 4, life-threatening symptoms, requiring emergency treatment.

### Statistical analysis

Taking α = 0.05, β = 0.10, and bilateral testing, the PASS software (version 11.0) was used to calculate the sample size in this study. Prognostic parameters were evaluated by multivariate Cox regression analysis. PFS, OS and CSS curves were obtained by the Kaplan–Meier method. Background characteristics were assessed by the t test, chi-square test or Kruskal–Wallis test, as appropriate. *P* < 0.05 indicated statistical significance. SPSS v20.0 (SPSS, USA) was employed for all statistical analyses.

## Results

### Patient features

**Figure 1:**
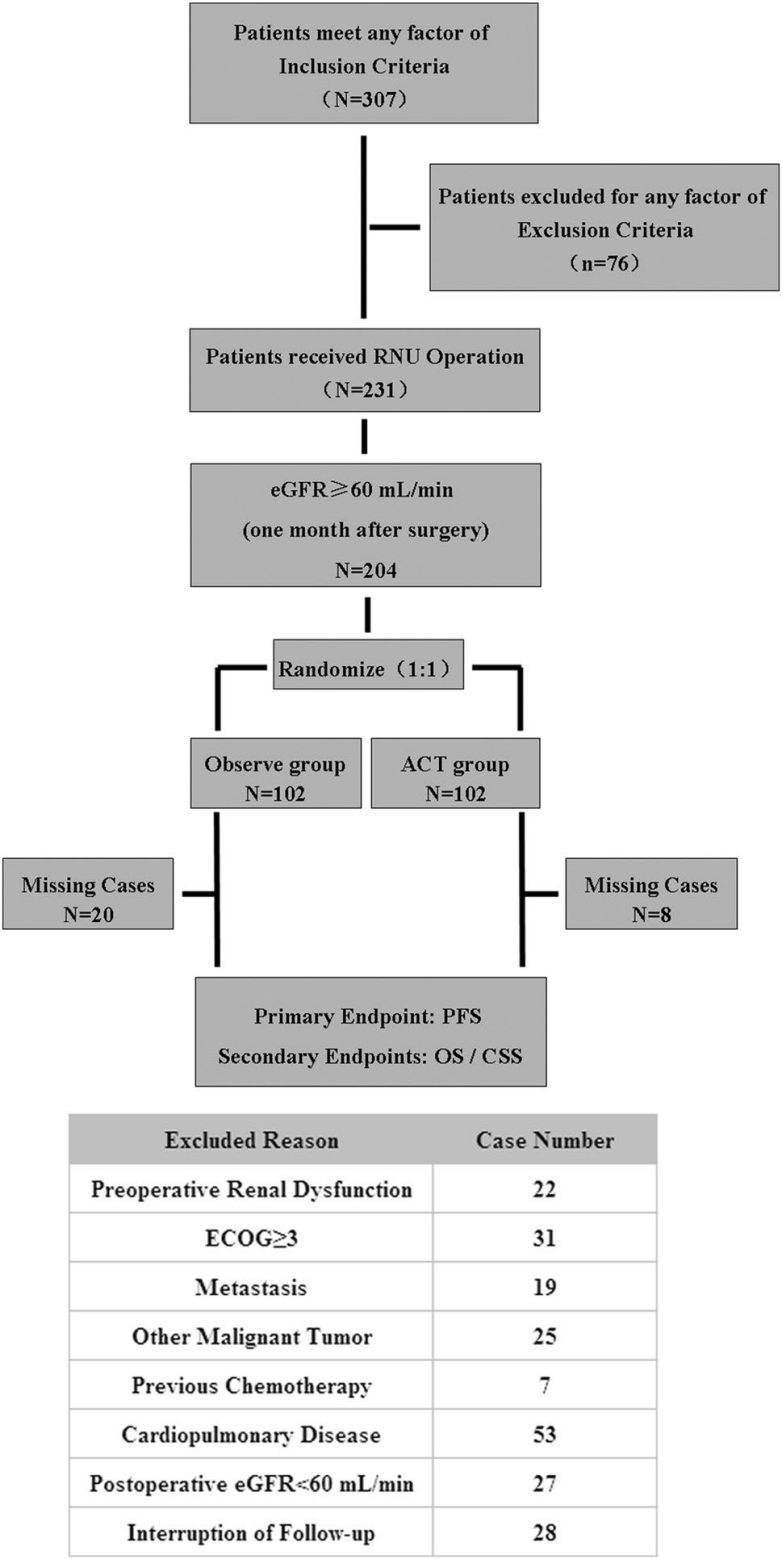
Patient selection flowchart.

A total of 307 patients with high-risk UTUC were assessed in the present study (Fig. 1). After 103 ineligible patients were excluded, the remaining 204 cases were randomized into two groups. During follow-up, totally 28 cases were lost. In the observation group, 20 patients withdrew from the study, because they lived far away from Beijing, and regular follow up was inconvenient. In the chemotherapy group, 8 patients dropped out of the study. Six of them were unable to complete the follow-up after the 3-cycle chemotherapy because of long distance from home, and another 2 voluntarily gave up treatment and follow up, because of eGFR decreasing to less than 60 mL/min (52 mL/min and 58 mL/min, respectively) after 2 cycles of chemotherapy.

Finally, 176 patients underwent the recommended treatment and active follow-up. Among these patients, 82 cases underwent RNU and 94 received RNU + ACT. The detailed clinicopathological features are shown in Table 1. There were no significant differences between the two groups in age at diagnosis, surgical technique, BMI, pathological stage, lymph node invasion, and tumor grade; only the amounts of operative bleeding significantly differed (*P* = 0.035).

**Table 1:** Detailed clinicopathological features of two treatment groups of high-risk UTUC patients.

Treatment	RNU (n = 82)	RNU + ACT (n = 94)	Statistic analysis	
	Median (range)	Median (range)	χ^2^	*P* value
Age at diagnosis (years)	67 (41–81)	64 (33–79)	1.626	0.331
Follow-up (months)	30 (6–54)	36 (6–54)	0.933	0.665

	Mean ± Standard	Mean ± Standard	T	*P* Value
Surgery time (min)	189 ± 38	171 ± 32	1.748	0.088
Surgery bleeding (mL)	241 ± 60	192 ± 87	2.183	0.035

	Count (%)	Count (%)	χ ^2^	*P* Value
**Gender**				
Male	31 (37.8)	37 (39.4)	0.045	0.832
Female	51 (62.2)	57 (60.6)		
**Surgical technique**				
Open	26 (31.7)	30 (31.9)	0.001	0.975
Laparoscopically	56 (68.3)	64 (68.1)		
**BMI**				
< 18.5	17	22	4.188	0.123
18.5–24	46	39		
≥ 24	19	33		
**ECOG**				
0–1	53 (64.6)	57 (60.6)	0.298	0.585
2	29 (35.4)	37 (39.4)		
**Pathological T stage**				
Ta/T1	8 (9.8)	6 (6.4)	2.744	0.433
T2	45 (54.9)	44 (46.8)		
T3	24 (29.3)	38 (40.4)		
T4	5 (6.1)	6 (6.4)		
**Lymph node involvement**				
Nx/N0	50 (61.0)	44 (46.8)	3.532	0.060
N+	32 (39.0)	50 (53.2)		
**Tumor grade**				
G1	24 (29.3)	28 (29.8)	0.183	0.913
G2	19 (23.2)	24 (25.5)		
G3	39 (47.5)	42 (44.7)		
**Tumor location**				
Renal pelvis	48 (58.5)	51 (54.2)	2.402	0.301
Ureter	28 (34.1)	40 (42.6)		
Both	6 (7.3)	3 (3.2)		
**CVE risk (ANZHEN)**				
Low risk	37 (45.1)	52 (55.3)	2.03	0.362
Intermediate risk	29 (35.4)	29 (30.9)		
High risk	16 (19.5)	13 (13.8)		
**Cardiovascular comorbidity**				
Coronary heart disease	39 (47.6)	42 (44.7)	3.224	0.521
Valvular heart disease	12 (14.6)	19 (20.2)		
Arrhythmia	7 (8.5)	10 (10.6)		
Aneurysm	11 (13.4)	15 (16.0)		
None	13 (15.9)	8 (8.5)		

UTUC, upper tract urothelial carcinoma; RNU, radical nephroureterectomy; ACT, adjuvant chemotherapy; BMI, body mass index; CVE, cardiovascular event.

### Multivariate analyses for survival prognosis

Table 2 summarizes multivariate analyses of predictors of survival outcomes. Multivariate analyses indicated that tumor size was independent prognostic indicator of PFS (*P* = 0.018) and CSS (*P* = 0.022), while BMI (*P* = 0.048) and LVI (*P* = 0.039) were indicators of PFS. In particular, peri-operative CVE risk was important independent predictor for OS (*P* = 0.031). More importantly, clinical stage, lymph node involvement, and tumor grade were significant predictors of PFS, OS and CSS in patients with high-risk UTUC. It should be emphasized that ACT had positive effects on PFS (*P* < 0.001), OS (*P* = 0, 013) and CSS (*P* = 0.008) after cardiovascular complications miscellaneous interference was excluded, despite the concern that cardiovascular comorbidity may have adverse confounding effects on the survival benefits of chemotherapy.

**Table 2:** Multivariable analyses for prognostic indicators of survival outcomes.

Variable	Progression free survival		Overall survival		Cancer Specific Survival	
	*P* value	HR (95% CI)	*P* value	HR (95% CI)	*P* value	HR (95% CI)
**Age at diagnosis (years)**						
≤ 65 versus > 65	0.135	–	0.119	–	0.093	–
**Surgical technique**						
Open versus laparoscopically	0.205	–	0.277	–	0.141	–
**BMI**						
< 18.5 versus 18.5–24 versus > 24	0.048	1.066 (1.012–1.098)	0.223	–	0.053	–
**CVE risk (ANZHEN)**						
Low versus intermediate versus high	0.532	–	0.031	1.294 (1.068–1.481)	0.286	–
**Tumor size**						
< 2 cm versus ≥ 2 cm	0.018	1.881 (1.562–2.041)	0.435	–	0.022	1.561 (1.076–1.858)
**Lymphovacular invasion**						
Positive versus negative	0.039	1.133 (1.008–1.205)	0.385	–	0.407	–
**Chemotherapy**						
Yes versus No	< 0.001	6.327 (5.115–7.793)	0.013	2.336 (1.956–2.883)	0.008	3.073 (2.533–3.738)
**Pathological T stage**						
≤ T2 versus ≥ T3	< 0.001	5.615 (4.823–6.326)	0.004	3.942 (3.254–4.823)	0.025	1.432 (1.153–1.703)
**Lymph node involvement**						
Nx/N0 versus N1	0.036	1.218 (1.034–1.502)	0.008	3.011 (2.264–3.755)	0.019	1.803 (1.632–1.974)
**Tumor grade**						
G1 versus G2 versus G3	0.003	4.322 (3.662–4.918)	0.027	1.418 (1.182–1.711)	0.007	3.223 (2.817–3.541)
**Tumor location**						
Renal pelvis versus ureter versus both	0.638	–	0.453	–	0.335	–

BMI, body mass index; HR, hazard ratio; CVE, cardiovascular event.

### ACT improves survival indexes in high-risk patients after RNU

**Figure 2:**
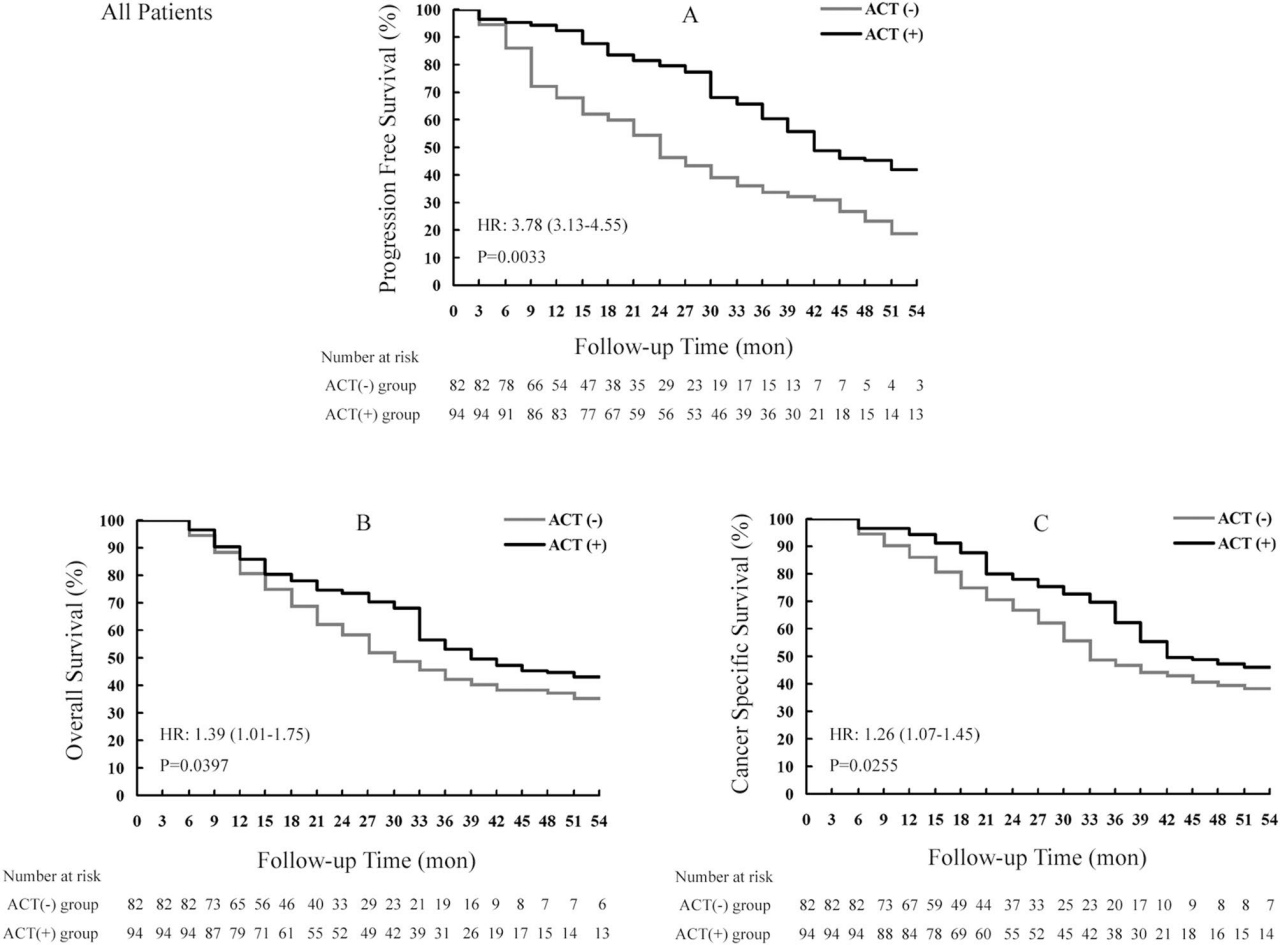
Kaplan–Meier curves for survival outcomes in all high-risk UTUC patients who underwent RNU surgery with or without the ACT strategy. UTUC, upper tract urothelial carcinoma; RNU, radical nephroureterectomy; ACT, adjuvant chemotherapy.

PFS rates between the two treatment regimens were compared. As shown in Fig. 2A, remarkably higher values were obtained in the RNU + ACT group in comparison with the RNU group at 12 months (72.4% vs. 94.2%), 24 months (54.7% vs. 81.6%), 36 months (36.2% vs. 65.6%), 48 months (27.1% vs. 46.3%), and 54 months (38.5% vs. 46.3%). The median PFS was starkly increased in the RNU + ACT group compared with the RNU group [25.5 months vs. 44.8 months, *P* = 0.0033, HR = 3.78 (3.13–4.55)].

As shown in Fig. 2B, OS rates in the RNU + ACT group were starkly elevated compared with those of the RNU group at 12 months (88.4% vs. 90.3%), 24 months (62.3% vs. 74.7%), 36 months (45.7% vs. 56.5%), 48 months (38.6% vs. 45.5%), and 54 months (35.2% vs. 43.1%). Median OS times were 32.7 and 41.8 months in patients administered RNU and RNU + ACT, respectively [*P* = 0.0397, HR = 1.39 (1.01–1.75)].

Figure 2C displays the trend of CSS rates, which were markedly elevated in the RNU + ACT group in comparison with the RNU group at 12 months (96.7% vs. 90.2%), 24 months (80.1% vs. 70.6%), 36 months (69.6% vs. 48.7%), 48 months (48.9% vs. 40.6%), and 54 months (46.3% vs. 38.5%). Meanwhile, the combination significantly increased the median time of CSS, from 34.2 months in the RNU group to 44.7 months in RNU + ACT treated patients [*P* = 0.0255, HR = 1.26 (1.07–1.45)].

### ACT improves survival indexes in RNU treated high-risk patients with lymph node metastasis

**Figure 3:**
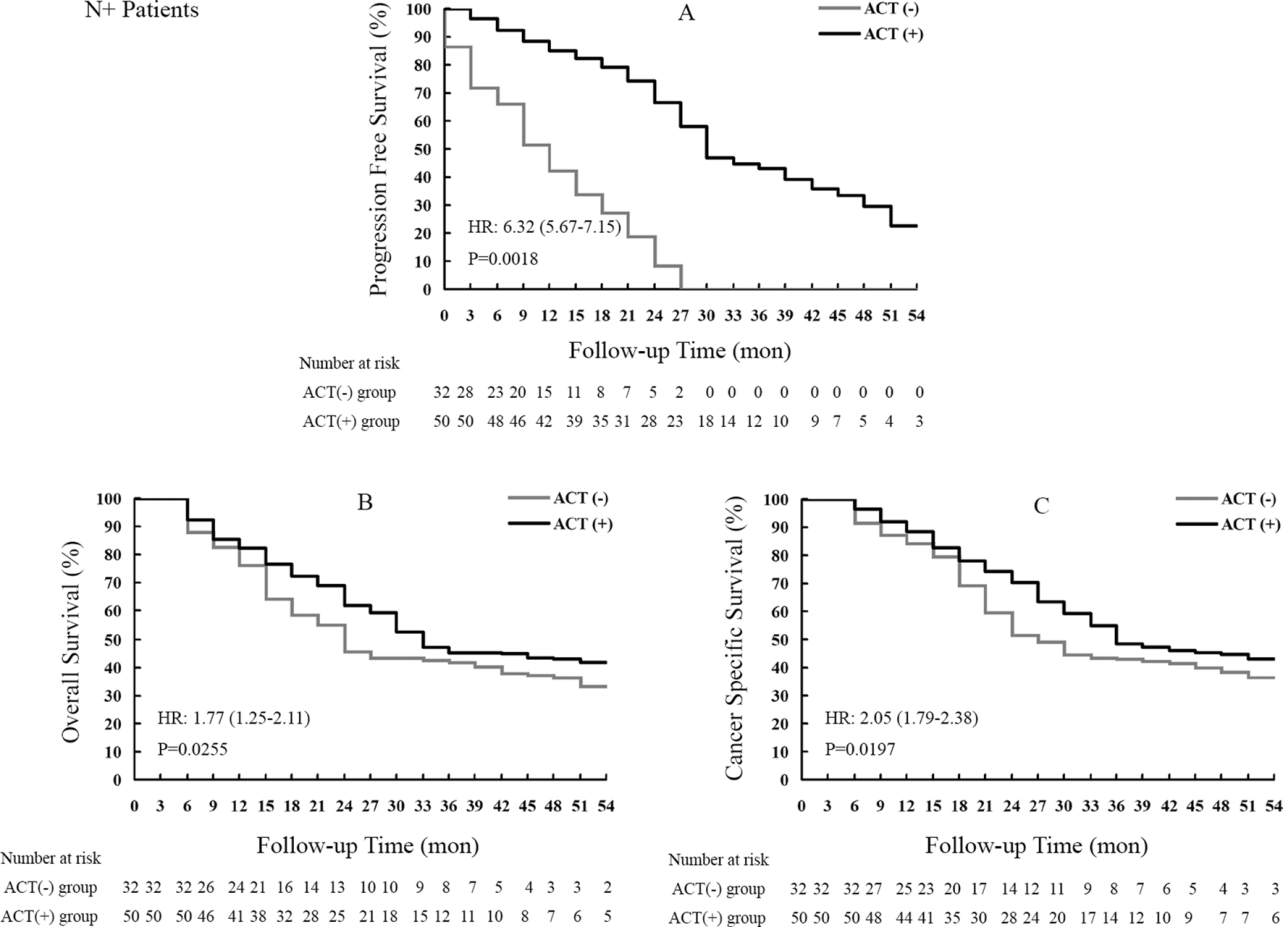
Kaplan–Meier curves for survival outcomes in lymph node invasive UTUC patients who underwent RNU surgery with or without the ACT strategy. UTUC, upper tract urothelial carcinoma; RNU, radical nephroureterectomy; ACT, adjuvant chemotherapy.

PFS was notably improved in RNU + ACT treated cases in comparison with the RNU group, as displayed in Fig. 3A. The median PFS time was significantly prolonged from 11.4 to 31.9 months [*P* = 0.0018, HR = 6.32 (5.67–7.15)] by the ACT strategy in high-risk cases who underwent RNU.

As shown in Fig. 3B, a median OS of 36.3 months was found in the RNU + ACT group, versus 26.8 months in RNU treated individuals, indicating a 9.5-month increase of OS [*P* = 0.0255, HR = 1.77 (1.25–2.11)].

The superiority of RNU + ACT over RNU was also shown in CSS (Fig. 3C). Indeed, median CSS times were 28.2 and 39.3 months in the RNU and RNU + ACT groups, respectively. The ACT based combination starkly increased the CSS rate in comparison with the RNU single treatment [*P* = 0.0197, HR = 2.05 (1.79–2.38)].

### ACT improves survival indexes in RNU treated high-risk patients with stage T3/T4 disease

**Figure 4:**
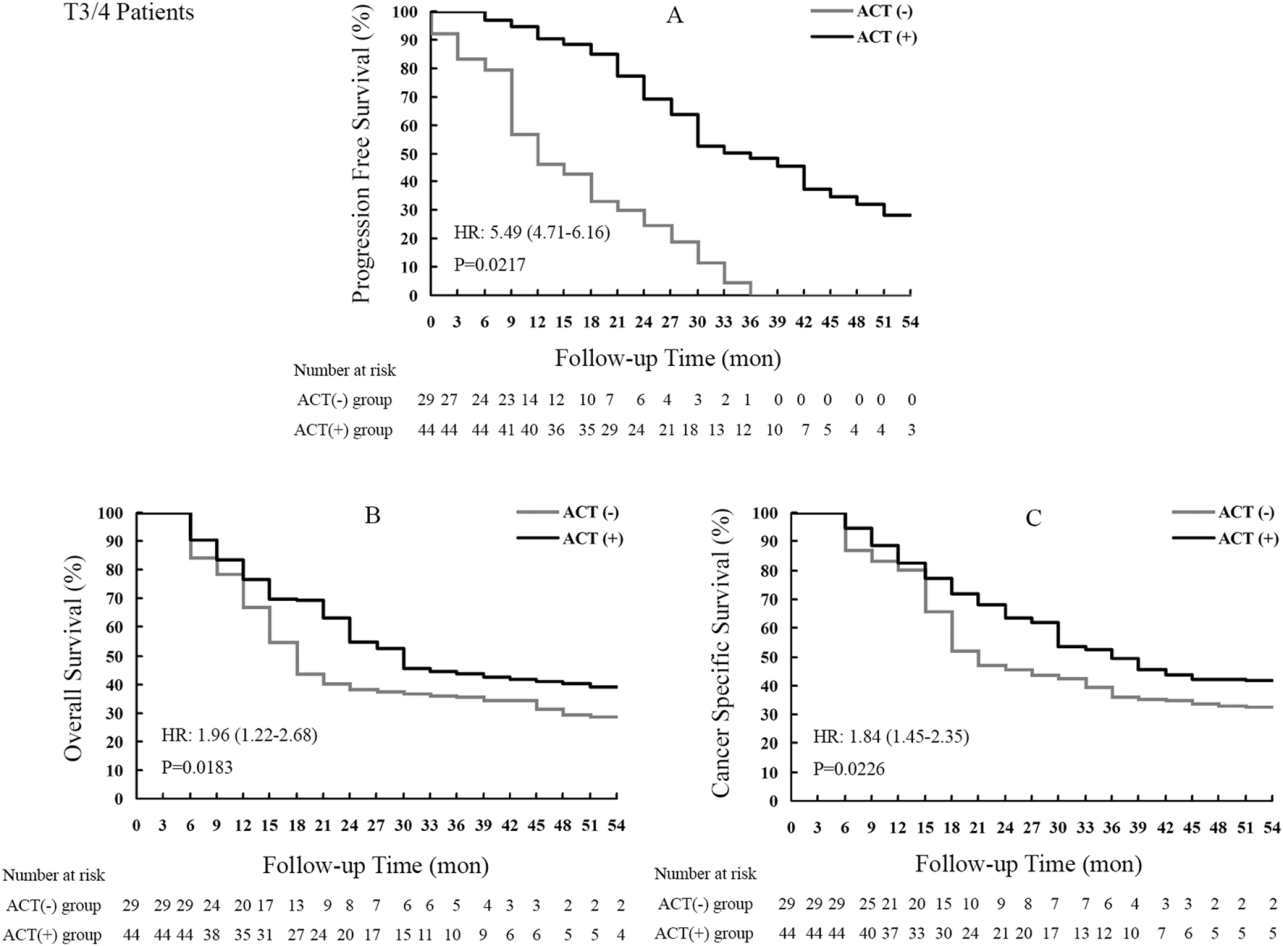
Kaplan–Meier curves for survival outcomes in T3/T4 stage UTUC patients who underwent RNU surgery with or without the ACT strategy. UTUC, upper tract urothelial carcinoma; RNU, radical nephroureterectomy; ACT, adjuvant chemotherapy.

PFS in RNU + ACT treated cases was notably improved compared with that of the RNU group, as displayed in Fig. 4A. The median PFS time was significantly prolonged from 13.9 to 36.3 months [*P* = 0.0217, HR = 5.49 (4.71–6.16)] by the ACT strategy in high-risk cases who underwent RNU.

As shown in Fig. 4B, a median OS of 32.2 months was found in the RNU + ACT group, versus 20.6 months in RNU treated individuals, indicating a 11.6-month increase of OS [*P* = 0.0183, HR = 1.96 (1.22–2.68)].

The superiority of RNU + ACT over RNU was equally observed in CSS (Fig. 4C). Indeed, median CSS durations were 21.9 and 38.4 months in the RNU and RNU + ACT groups, respectively. In addition, the ACT based combination markedly increased the CSS rate in comparison with the RNU single treatment [*P* = 0.0226, HR = 1.84 (1.45–2.35)].

### Complications in ACT treated high-risk patients

All chemotherapy related complications are shown in Table 3. Medullary hematopoiesis function, skin and mucosal system functions, endocrine symptoms, digestive function, cardiovascular events and several important organ functions were carefully evaluated and detailedly recorded.

**Table 3:** Summary of cytotoxic chemotherapy related complications of all high-risk UTUC patients underwent ACT strategy.

Toxic complications	Grade 1 count (%)	Grade 2 count (%)	Grade 3 count (%)	Grade 4 count (%)
**Hematological system**				
Leucocyte	36 (38.3)	9 (9.6)	2 (2.1)	0
Granulocyte	31 (33.0)	13 (13.8)	2 (2.1)	0
Hemoglobin	18 (19.1)	8 (8.5)	2 (2.1)	0
Platelet	7 (7.4)	4 (4.3)	1 (1.1)	0
**Urinary system**				
Blood urea nitrogen	19 (20.2)	11 (11.7)	0	0
Serum creatinine	34 (36.2)	6 (6.4)	0	0
Hematuria	26 (27.7)	3 (3.2)	0	0
**Digestive system**				
Nausea/vomiting	21 (22.3)	12 (12.8)	19 (20.2)	0
Diarrhea	9 (9.6)	5 (5.3)	0	0
Constipation	41 (43.6)	21 (22.3)	1 (1.1)	0
Alanine transarninase	13 (13.8)	8 (8.5)	0	0
Alkaline phosphatase	9 (9.6)	3 (3.2)	0	0
Bilirubin	12 (12.8)	2 (2.1)	0	0
**Skin/oral mucosa**				
Ulcer	11 (11.7)	6 (6.4)	3 (3.2)	0
Erythema/pruritus	15 (16.0)	15 (16.0)	1 (1.1)	0
Herpes	13 (13.8)	3 (3.2)	2 (2.1)	0
Baldness	9 (9.6)	5 (5.3)	1 (1.1)	0
Phlebitis	8 (8.5)	1 (1.1)	1 (1.1)	0
**Cardiovascular system**				
Arrhythmia	7 (7.4)	2 (2.1)	0	0
Cardiac dysfunction	5 (5.3)	8 (8.5)	1 (1.1)	0
Pericarditis	1 (1.1)	1 (1.1)	0	0

All chemotherapy related symptoms were evaluated according to CACTE 4.0 toxicity scoring criteria; UTUC, upper tract urothelial carcinoma; ACT, adjuvant chemotherapy.

Most complications were distributed in the medullary hematopoiesis and digestive systems, and mainly included grade 1 and 2 events. Twenty-eight patients (29.8%) showed grade 3 complications. Among them, three cases displayed severely decreased amounts of neutrophilic granulocytes, hemoglobin and leucocytes; meanwhile, twenty cases showed severe nausea, vomiting or constipation. Four cases were further treated for oral ulcers, phlebitis or herpes, and one case developed acute cardiac dysfunction. No patients displayed severe (grade 4) complications. All these symptoms could be improved with medication. In addition, forty patients (42.6%) were diagnosed with grade 1 and 2 renal function injury, not requiring active intervention; conservative measures were generally effective in such cases. The RNU + ACT regimen caused no serious complications.

## Discussion

Based on several retrospective studies, adjuvant chemotherapy does not exert convincing effects on survival advantage in UTUC cases. Hellenthal et al.^5^ comparatively analyzed 542 high-risk UTUC cases retrospectively, and found no remarkable differences in OS (HR = 1.06, *P* = 0.687) and CSS (HR = 1.26, *P* = 0.129) between cases treated by the ACT strategy and those who received no ACT. Soga et al.^6^ retrospectively evaluated 132 high-risk patients who underwent RNU, and the 10-year survival outcome was not improved by ACT (95.8% vs. 86.5%, *P* = 0.081); however, disease recurrence in the bladder was significantly decreased in the ACT group (*P* < 0.0001). Cohen et al.^7^ retrospectively assessed 3432 UTUC cases with a 5-year follow-up, including 11.8% of all patients treated with RNU plus ACT; compared with cases administered RNU alone, the patients that received RNU + ACT showed no improvement in the 5-year CSS outcome. Kim et al.^8^ also assessed 65 patients with high-risk UTUC, of whom 36 cases underwent RNU alone and 29 were treated with RNU plus cisplatin chemotherapy. After 34 months of follow-up, RNU + ACT was shown to be significantly more beneficial compared with the RNU monotherapy in bladder RFS (41.4% vs. 13.9%, *P* = 0.001), but not improving CSS. Another study also reported no remarkable differences between the adjuvant and non-adjuvant groups in RFS (*P* = 0.794) and CSS (*P* = 0.783) in 40 months of follow-up^9^. Recently, a retrospective trial^10^ by the European Association of Urology-Young Academic Urologists (EAU-YAU) also showed that adjuvant chemotherapy does not increase OS in comparison with observation in high-risk post-operation patients (HR = 1.14, *P* = 0.268).

However, findings reported by several other studies remain controversial. Seisen et al.^11^ reported that median OS was markedly prolonged by ACT compared with observation (47.41 months vs. 35.78 months, *P* < 0.001) in 3253 individuals with high-risk UTUC administered RNU. Meanwhile, 5-year OS rates in the ACT and observation groups were 43.90% and 35.85%, respectively (*P* < 0.001). In addition, Lee et al.^12^ comparatively analyzed 344 UTUC patients with or without LVI, and reported that ACT after surgery could independently improve survival in patients with LVI, in terms of CSS (HR = 0.51, *P* = 0.027) and OS (HR = 0.50, *P* = 0.025), without affecting survival in cases without LVI. Further stratified analysis by pathological stage indicated that ACT was significantly beneficial for survival in T3/T4 cases in terms of CSS (HR = 0.39, *P* = 0.028) and OS (HR = 0.410, *P* = 0.031), but not in T1/T2 cases. Furthermore, Huang et al.^13^ retrospectively evaluated the detailed data of 171 cases with T3N0M0 treated by RNU, and found statistically significant differences in CSS (80.5% vs. 57.6%, *P* = 0.010) and RFS (74.4% vs. 52.9%, *P* = 0.026) between the adjuvant and non-adjuvant treatment groups during a 5-year follow-up period. A recent multi-institutional retrospective study^14^ found that cisplatin-based ACT is tightly associated with improved RFS (HR = 0.41; *P* = 0.0178) and CSS (HR = 0.33; *P* = 0.0375) in multivariate analysis.

Contrastively analyzing most of these published studies, we found that the survival effect of ACT may be influenced by multiple pathological factors, such as high tumor staging and positive LVI. It is very difficult to balance the selective biases in these retrospective trials; therefore, the conclusion about the benefit of ACT may be inaccurate, and remains uncertain. Recently, the partial results of the POUT study^15^, which was designed as a prospective randomized trial to compare oncologic outcome between peri-operative chemotherapy and surveillance in UTUC patients, were publicly reported in the ASCO-GU’s annual meeting, demonstrating that ACT could remarkably improve disease-free survival (DFS) in high-risk UTUC patients administered RNU (HR = 0.49, *P* = 0.001); the DFS rate was raised from 43.1% to 72.5% by ACT treatment at 36 months of follow-up. However, a difference in OS has not been observed to date (HR = 0.55).

We specially performed this prospective randomized controlled trial to accurately evaluate the utility of ACT in high-risk UTUC patients undergoing the RNU procedure. In this study, 176 patients with high-risk UTUC were selected and followed up to observe the improvement effect of ACT treatment on patient survival after radical surgery. However, the included patients were not ordinary UTUC cases. Eighty-eight percent of them had different degrees and types of cardiac or macrovascular comorbidities, and some underwent percutaneous coronary intervention (PCI), coronary artery bypass grafting (CABG), cardiac pacemaker implantation, radiofrequency ablation, aortic replacement or stent implantation, total arch artery replacement, et al. In addition, others had mild cardiovascular comorbidity without cardiovascular surgical treatment.

Before initiation of the study, the potential cardiotoxicity of chemotherapy in affecting the cardiovascular function and even the survival outcomes of patients was not concerned. Therefore, during chemotherapy, we divided the dose of cisplatin to reduce the toxicity of chemotherapy. Meanwhile, the adjuvant chemotherapy was temporarily set as three courses. Through such adjustment, we tried to explore whether adjuvant chemotherapy can reflect the real trend of improving survival and prognosis, and whether it has certain safety, so as to lay some preliminary treatment experience for the later stage of adjuvant chemotherapy. Finally, from data of all patients with cardiovascular complications, we found that chemotherapy significantly predicted improved PFS and CSS in high-risk UTUC patients after radical surgery, instead of exerted adverse effects on OS. Therefore, we further sorted out and stratified the treatment risk of cardiovascular complications in all patients, and re-conducted multivariable analysis according to the new CVE risk assessment criteria. After eliminating the confounding interference of cardiovascular complications, ACT showed a positive improvement value for OS.

Chemotherapy not only significantly improve oncological outcomes in patients with cardiovascular comorbidity, more importantly, in the included patients, after 3 cycles of chemotherapy, the risk of disease progression was significantly reduced by 3.78 fold; this risk in patients with positive lymph nodes and high-stage cases was decreased by 6.32 and 5.49 fold, respectively. The risk of overall mortality was significantly reduced by 1.39 times, with 1.77- and 1.96-fold reduction in patients with positive lymph nodes and high-stage cases, respectively. The risk of tumor-specific death was significantly reduced by 1.26 times, with 2.05- and 1.84-fold reductions in patients with positive lymph nodes and high-stage cases, respectively. In addition, we further demonstrated that ACT could significantly increase the 54-month CSS, OS and PFS of high-risk UTUC patients, especially in cases with T3/4 stage disease or lymph node metastasis. Chemotherapy-based combined therapy was essential for OS and CSS improvement in high-risk UTUC cases; and time to radiographic progression was also relatively prolonged. These clinical findings comprehensively revealed that post-operative chemotherapy markedly controls tumor progression and improves survival in high-risk UTUC patients, even if with cardiovascular comorbidity.

Till now, there is no definite consensus regarding the actual effect of chemotherapy on solitary renal function. A retrospective study reviewed the changing trends of renal function in 183 advanced UTUC cases administered the RNU surgery^16^. Among these patients, 122 (66.7%) received cisplatin-based chemotherapy. The comparison data showed that serum creatinine levels and eGFR were comparable before and after chemotherapy (CREA, 102.7 ± 32.8 μmol/L vs. 106.0 ± 49.6 μmol/L, *P* = 0.294; eGFR, 65.8 ± 20.8 mL/min vs. 65.8 ± 21.8 mL/min, *P* > 0.999). In the multivariate logistic regression model, cisplatin-based chemotherapy did not impair renal function (OR = 0.95, *P* = 0.893); over 4 cycles of first-line chemotherapy may increase the risk of renal dysfunction, but the difference was not significant (OR = 1.41, *P* = 0.398). Additionally, the POUT study also revealed no severe renal function damage above grade 3 in3 years of follow-up^15^. In present study, we found that the scheme of cisplatin divided administration can not only effectively control the cardiovascular toxicity, but also maximally reduce the damage to renal function. If eGFR of patients was over 60 mL/min, post-operative chemotherapy showed safe treatment effect. Whether patients with serious renal dysfunction could receive chemotherapy after surgery still requires deep investigation.

However, this current study had some limitations, including a single-center trial design, small cohort size, many involved surgeons, and the variability of intra-operative management. In future study, we will cooperate with other medical centers to enroll more high-risk patients without other complications, prolong the follow-up time and better match the background characteristics of patients. During study, patients with cardiovascular complications and patients without complications were followed up and observed respectively, and special designated surgeons were determined to avoid the increase of intraoperative emergencies due to complications, and to obtain more objective and accurate conclusions. At the same time, increasing the cycle of chemotherapy to further observe whether the improvement of the prognosis of patients with chemotherapy has a change trend should also be performed. In addition, in the current clinical practice, due to the potential heart and kidney toxicities of adjuvant chemotherapy, most patients with severe heart and renal insufficiencies do not receive adjuvant chemotherapy after radical operation. Therefore, in further investigation, high-risk UTUC patients with eGFR < 60 ml/min or very high risk of CVE will be included, and administered a GC regimen with half-dose, in order to evaluate the safety and effectiveness of adjuvant chemotherapy in such patients.

## Conclusion

In all, ACT treatment is a safe and effective strategy for post-operation in high-risk UTUC cases with cardiovascular comorbidity. Specifically, ACT treatment could significantly prolong median survival time in individuals with lymph node metastasis and T3/T4 stage cases. Additionally, no patients displayed severe complications.

## Supplementary information

Supplementary Information 1.

Supplementary Information 2.

Supplementary Information 3.

## References

[1] Leow JJ, Chong KT, Chang SL, Bellmunt J (2017). Upper tract urothelial carcinoma: a different disease entity in terms of management. ESMO Open.

[2] Kubota Y (2017). Oncological outcomes of neoadjuvant chemotherapy in patients with locally advanced upper tract urothelial carcinoma: a multicenter study. Oncotarget.

[3] Aziz A (2017). Perioperative chemotherapy in upper tract urothelial carcinoma: a comprehensive review. World J. Urol..

[4] Azizi M (2019). Optimal management of upper tract urothelial carcinoma: an unmet need. Curr. Treat. Options Oncol..

[5] Hellenthal NJ (2009). Adjuvant chemotherapy for high risk upper tract urothelial carcinoma: results from the Upper Tract Urothelial Carcinoma Collaboration. J. Urol..

[6] Soga N, Arima K, Sugimura Y (2010). Adjuvant methotrexate, vinblastine, adriamycin, and cisplatin chemotherapy has potential to prevent recurrence of bladder tumors after surgical removal of upper urinary tract transitional cell carcinoma. Int. J. Urol..

[7] Cohen A, Kuchta K, Park S (2017). Neoadjuvant and adjuvant chemotherapy use in upper tract urothelial carcinoma. Urol. Oncol..

[8] Kim TS, Oh JH, Rhew HY (2013). The efficacy of adjuvant chemotherapy for locally advanced upper tract urothelial cell carcinoma. J. Cancer.

[9] Lee SE (2006). Adjuvant chemotherapy in the management of pT3N0M0 transitional cell carcinoma of the upper urinary tract. Urol. Int..

[10] Necchi A (2018). Adjuvant chemotherapy after radical nephroureterectomy does not improve survival in patients with upper tract urothelial carcinoma: a joint study by the European Association of Urology-Young Academic Urologists and the Upper Tract Urothelial Carcinoma Collaboration. BJU Int..

[11] Seisen T (2017). Effectiveness of adjuvant chemotherapy after radical nephroureterectomy for locally advanced and/or positive regional lymph node upper tract urothelial carcinoma. J. Clin. Oncol..

[12] Lee KS (2015). Impact of adjuvant chemotherapy in patients with upper tract urothelial carcinoma and lymphovascular invasion after radical nephroureterectomy. Korean J. Urol..

[13] Huang YC (2015). The efficacy of postoperative adjuvant chemotherapy for patients with pT3N0M0 upper tract urothelial carcinoma. J. Urol..

[14] Nakagawa T (2017). Efficacy of post-nephroureterectomy cisplatin-based adjuvant chemotherapy for locally advanced upper tract urothelial carcinoma: a multi-institutional retrospective study. World J. Urol..

[15] Birtle AJ, Chester JD, Jones RJM (2018). Results of POUT: A phase III randomised trial of perioperative chemotherapy versus surveillance in upper tract urothelial cancer (UTUC). J. Clin. Oncol..

[16] Li X (2018). Impact of first-line chemotherapy on renal function in patients with advanced upper tract urothelial carcinoma. Zhonghua Yi Xue Za Zhi.

