# Supplementary material for: Adjuvant chemotherapy after radical nephroureterectomy improves the survival outcome of high-risk upper tract urothelial carcinoma patients with cardiovascular comorbidity
Source: Sci Rep. 2020 Oct 19;10:17674. doi: 10.1038/s41598-020-74940-x (PMC7572393; doi:10.1038/s41598-020-74940-x)
Supplement: Supplementary file 1 — Supplementary Information 1. [file 41598_2020_74940_MOESM1_ESM.doc]

**Adjuvant chemotherapy after radical nephroureterectomy improves the survival outcome of high-risk upper tract urothelial carcinoma patients with cardiovascular comorbidity**

**LUO Yong*, FENG Bingfu, WEI Dechao, HAN Yili, LI Mingchuan, ZHAO Jiahui, LIN Yunhua, HOU Zhu, JIANG Yongguang**

**Department of Urology, Beijing Anzhen Hospital, Capital Medical University. Anzhenli Street, Chaoyang District, Beijing, 100029, PR. China.**

**Protocol Number: < Number>**

**National Clinical Trial (NCT) Identified Number: < ChiCTR1900027924 >**

**Principal Investigator:** **< Yong Luo >**

**<IND/IDE> Sponsor: < Beijing Natural Science Foundation >Funded by: < Beijing Natural Science Foundation>**

**Version Number: v.<x.x>**

**<01 December 2019 >**

**Summary of Changes from Previous Version:**

| **Affected Section(s)** | **Summary of Revisions Made** | **Rationale** |
| --- | --- | --- |
|  |  |  |
|  |  |  |

Table of Contents

[STATEMENT OF COMPLIANCE 3](#__RefHeading___Toc479192697)

[1 PROTOCOL SUMMARY 3](#__RefHeading___Toc479192698)

[1.1 Synopsis 3](#__RefHeading___Toc479192699)

[1.2 Schema 3](#__RefHeading___Toc479192700)

[1.3 Schedule of Activities (SoA) 3](#__RefHeading___Toc479192701)

[2 INTRODUCTION 3](#__RefHeading___Toc479192702)

[2.1 Study Rationale 3](#__RefHeading___Toc479192703)

[2.2 Background 3](#__RefHeading___Toc479192704)

[2.3 Risk/Benefit Assessment 3](#__RefHeading___Toc479192705)

[2.3.1 Known Potential Risks 3](#__RefHeading___Toc479192706)

[2.3.2 Known Potential Benefits 3](#__RefHeading___Toc479192707)

[2.3.3 Assessment of Potential Risks and Benefits 3](#__RefHeading___Toc479192708)

[3 OBJECTIVES AND ENDPOINTS 3](#__RefHeading___Toc479192709)

[4 STUDY DESIGN 3](#__RefHeading___Toc479192710)

[4.1 Overall Design 3](#__RefHeading___Toc479192711)

[4.2 Scientific Rationale for Study Design 3](#__RefHeading___Toc479192712)

[4.3 Justification for Dose 3](#__RefHeading___Toc479192713)

[4.4 End of Study Definition 3](#__RefHeading___Toc479192714)

[5 STUDY POPULATION 3](#__RefHeading___Toc479192715)

[5.1 Inclusion Criteria 3](#__RefHeading___Toc479192716)

[5.2 Exclusion Criteria 3](#__RefHeading___Toc479192717)

[5.3 Lifestyle Considerations 3](#__RefHeading___Toc479192718)

[5.4 Screen Failures 3](#__RefHeading___Toc479192719)

[5.5 Strategies for Recruitment and Retention 3](#__RefHeading___Toc479192720)

[6 STUDY INTERVENTION 3](#__RefHeading___Toc479192721)

[6.1 Study Intervention(s) Administration 3](#__RefHeading___Toc479192722)

[6.1.1 Study Intervention Description 3](#__RefHeading___Toc479192723)

[6.1.2 Dosing and Administration 3](#__RefHeading___Toc479192724)

[6.2 Preparation/Handling/Storage/Accountability 3](#__RefHeading___Toc479192725)

[6.2.1 Acquisition and accountability 3](#__RefHeading___Toc479192726)

[6.2.2 Formulation, Appearance, Packaging, and Labeling 3](#__RefHeading___Toc479192727)

[6.2.3 Product Storage and Stability 3](#__RefHeading___Toc479192728)

[6.2.4 Preparation 3](#__RefHeading___Toc479192729)

[6.3 Measures to Minimize Bias: Randomization and Blinding 3](#__RefHeading___Toc479192730)

[6.4 Study Intervention Compliance 3](#__RefHeading___Toc479192731)

[6.5 Concomitant Therapy 3](#__RefHeading___Toc479192732)

[6.5.1 Rescue Medicine 3](#__RefHeading___Toc479192733)

[7 STUDY INTERVENTION DISCONTINUATION AND PARTICIPANT DISCONTINUATION/WITHDRAWAL 3](#__RefHeading___Toc479192734)

[7.1 Discontinuation of Study Intervention 3](#__RefHeading___Toc479192735)

[7.2 Participant Discontinuation/Withdrawal from the Study 3](#__RefHeading___Toc479192736)

[7.3 Lost to Follow-Up 3](#__RefHeading___Toc479192737)

[8 STUDY ASSESSMENTS AND PROCEDURES 3](#__RefHeading___Toc479192738)

[8.1 Efficacy Assessments 3](#__RefHeading___Toc479192739)

[8.2 Safety and Other Assessments 3](#__RefHeading___Toc479192740)

[8.3 Adverse Events and Serious Adverse Events 3](#__RefHeading___Toc479192741)

[8.3.1 Definition of Adverse Events (AE) 3](#__RefHeading___Toc479192742)

[8.3.2 Definition of Serious Adverse Events (SAE) 3](#__RefHeading___Toc479192743)

[8.3.3 Classification of an Adverse Event 3](#__RefHeading___Toc479192744)

[8.3.4 Time Period and Frequency for Event Assessment and Follow-Up 3](#__RefHeading___Toc479192745)

[8.3.5 Adverse Event Reporting 3](#__RefHeading___Toc479192746)

[8.3.6 Serious Adverse Event Reporting 3](#__RefHeading___Toc479192747)

[8.3.7 Reporting Events to Participants 3](#__RefHeading___Toc479192748)

[8.3.8 Events of Special Interest 3](#__RefHeading___Toc479192749)

[8.3.9 Reporting of Pregnancy 3](#__RefHeading___Toc479192750)

[8.4 Unanticipated Problems 3](#__RefHeading___Toc479192751)

[8.4.1 Definition of Unanticipated Problems (UP) 3](#__RefHeading___Toc479192752)

[8.4.2 Unanticipated Problem Reporting 3](#__RefHeading___Toc479192753)

[8.4.3 Reporting Unanticipated Problems to Participants 3](#__RefHeading___Toc479192754)

[9 STATISTICAL CONSIDERATIONS 3](#__RefHeading___Toc479192755)

[9.1 Statistical Hypotheses 3](#__RefHeading___Toc479192756)

[9.2 Sample Size Determination 3](#__RefHeading___Toc479192757)

[9.3 Populations for Analyses 3](#__RefHeading___Toc479192758)

[9.4 Statistical Analyses 3](#__RefHeading___Toc479192759)

[9.4.1 General Approach 3](#__RefHeading___Toc479192760)

[9.4.2 Analysis of the Primary Efficacy Endpoint(s) 3](#__RefHeading___Toc479192761)

[9.4.3 Analysis of the Secondary Endpoint(s) 3](#__RefHeading___Toc479192762)

[9.4.4 Safety Analyses 3](#__RefHeading___Toc479192763)

[9.4.5 Baseline Descriptive Statistics 3](#__RefHeading___Toc479192764)

[9.4.6 Planned Interim Analyses 3](#__RefHeading___Toc479192765)

[9.4.7 Sub-Group Analyses 3](#__RefHeading___Toc479192766)

[9.4.8 Tabulation of Individual participant Data 3](#__RefHeading___Toc479192767)

[9.4.9 Exploratory Analyses 3](#__RefHeading___Toc479192768)

[10 SUPPORTING DOCUMENTATION AND OPERATIONAL CONSIDERATIONS 3](#__RefHeading___Toc479192769)

[10.1 Regulatory, Ethical, and Study Oversight Considerations 3](#__RefHeading___Toc479192770)

[10.1.1 Informed Consent Process 3](#__RefHeading___Toc479192771)

[10.1.2 Study Discontinuation and Closure 3](#__RefHeading___Toc479192772)

[10.1.3 Confidentiality and Privacy 3](#__RefHeading___Toc479192773)

[10.1.4 Future Use of Stored Specimens and Data 3](#__RefHeading___Toc479192774)

[10.1.5 Key Roles and Study Governance 3](#__RefHeading___Toc479192775)

[10.1.6 Safety Oversight 3](#__RefHeading___Toc479192776)

[10.1.7 Clinical Monitoring 3](#__RefHeading___Toc479192777)

[10.1.8 Quality Assurance and Quality Control 3](#__RefHeading___Toc479192778)

[10.1.9 Data Handling and Record Keeping 3](#__RefHeading___Toc479192779)

[10.1.10 Protocol Deviations 3](#__RefHeading___Toc479192780)

[10.1.11 Publication and Data Sharing Policy 3](#__RefHeading___Toc479192781)

[10.1.12 Conflict of Interest Policy 3](#__RefHeading___Toc479192782)

[10.2 Additional Considerations 3](#__RefHeading___Toc479192783)

[10.3 Abbreviations 3](#__RefHeading___Toc479192784)

[10.4 Protocol Amendment History 3](#__RefHeading___Toc479192785)

[11 REFERENCES 3](#__RefHeading___Toc479192786)

# STATEMENT OF COMPLIANCE

1. [The trial will be carried out in accordance with International Conference on Harmonisation Good Clinical Practice (ICH GCP) and the following:

- United States (US) Code of Federal Regulations (CFR) applicable to clinical studies (45 CFR Part 46, 21 CFR Part 50, 21 CFR Part 56, 21 CFR Part 312, and/or 21 CFR Part 812)

National Institutes of Health (NIH)-funded investigators and clinical trial site staff who are responsible for the conduct, management, or oversight of NIH-funded clinical trials have completed Human Subjects Protection and ICH GCP Training.

The protocol, informed consent form(s), recruitment materials, and all participant materials will be submitted to the Institutional Review Board (IRB) for review and approval. Approval of both the protocol and the consent form must be obtained before any participant is enrolled. Any amendment to the protocol will require review and approval by the IRB before the changes are implemented to the study. In addition, all changes to the consent form will be IRB-approved; a determination will be made regarding whether a new consent needs to be obtained from participants who provided consent, using a previously approved consent form.]

# PROTOCOL SUMMARY

## Synopsis

| **Title:** | Oncologic outcomes of high·risk upper tract urothelial carcinoma patients undergoing radical nephrouretercctomy combined with adjuvant chemotherapy |
| --- | --- |
| **Study Description:** | 1. To assess the oncologic outcomes of radical nephroureterectomy combined with adjuvant chemotherapy(ACT)in patients with high risk upper tract urothelial carcinoma. One-hundred-thirty-four individuals with high-risk UTUC who underwent RUN with or without ACT were evaluated．Cox proportional hazard model and Kaplan-Meier analysis were used to determine overall and cancer specific survival in the cohort． |
| **Objectives:** | To assess the oncologic outcomes of radical nephroureterectomy combined with adjuvant chemotherapy in patients with high risk upper tract urothelial carcinoma |
|  | <Primary Objective: |
|  | Secondary Objectives: > |
| **Endpoints:** | 1. PFS (time to radiographic progression)   OS (time to death regardless of cause)  CSS (time elapsed from RNU to cancer death)  <Primary Endpoint: PFS (time to radiographic progression)  Secondary Endpoints: OS (time to death regardless of cause)  CSS (time elapsed from RNU to cancer death) |
| **Study Population:** | 1. 182 high-risk UTUC patients, |
| **Phase:** | Not applicable. |
| **Description of Sites/Facilities Enrolling Participants:** | Not applicable |
| **Description of Study Intervention:** | High-risk UTUC patients were randomly divided into two groups: RNU treatment group and RNU + ACT treatment group. Among the patients in the RNU group, only radical surgery was performed. The RUN + ACT group started three cycles of gemcitabine (Eli Lilly and Company, USA) and cisplatin (Qilu Pharmaceutical Co., Ltd., China) 3 months after the radical operation Weekly chemotherapy regimen (Gemcitabine 1,000 mg / m2 intravenously infusion on day 1 and 8; Cisplatin 70 mg / m2 intravenously in divided doses on day 2, 3 and 4) |
| **Study Duration:** | 50 months |
| **Participant Duration:** | 2014-2019 |
|  |  |

## Schema

Prior to

High-risk UTUC patients

Enrollment

Randomize

Blood routine and biochemical examination, In addition, in patients with ACT,

Chemotherapy Toxicity Scale.

Visit 1

Three months

Blood routine and biochemical examination, chest and abdomen CT review. In addition, in patients with ACT,

Chemotherapy Toxicity Scale.

Visit 2

Six months

Blood routine and biochemical examination, In addition, in patients with ACT,

Chemotherapy Toxicity Scale.

Visit 3

Nine months

Blood routine and biochemical examination, chest and abdomen CT review, In addition, in patients with ACT, Chemotherapy Toxicity Scale.

Visit 4

One year

Blood routine and biochemical examination, chest and abdomen CT review, In addition, in patients with ACT, Chemotherapy Toxicity Scale.

Visit 5

Eighteen

months

Blood routine and biochemical examination, chest and abdomen CT review, In addition, in patients with ACT, Chemotherapy Toxicity Scale.

Visit 6

Two years

PFS (time to radiographic progression)

OS (time to death regardless of cause)

CSS (time elapsed from RNU to cancer death)

## Schedule of Activities (SoA)

| **Procedures** | Screening  Day -7 to -1 | Enrollment/Baseline  Visit 1, Day 90 | Study Visit 2  Day 180 +/-7 day | Study Visit 3  Day 270 +/- 7 day | Study Visit 4  Day 360 +/-7 day | Study Visit 5  Day 540 +/-7 day | Study Visit 6  Day 720 +/-7 day |
| --- | --- | --- | --- | --- | --- | --- | --- |
| Informed consent | X |  |  |  |  |  |  |
| Demographics | X |  |  |  |  |  |  |
| Medical history | X |  |  |  |  |  |  |
| Randomization | X |  |  |  |  |  |  |
| Administer study intervention |  | X |  |  | X |  |  |
| Concomitant medication review | X |  | | | | | |
| Physical exam (including height and weight) | X | X |  |  | X |  |  |
| Vital signs | X | X |  |  | X |  |  |
| Height | X |  |  |  |  |  |  |
| Weight | X | X |  | X |  | X |  |
| Performance status | X | X |  | X |  | X |  |
| Hematology | X | X | X | X | X | X | X |
| serum chemistry ^a^ | X | X | X | X | X | X | X |
| Pregnancy test ^b^ | X |  |  |  |  |  |  |
| EKG (as indicated) | X |  |  |  |  |  |  |
| Adverse event review and evaluation | X |  | | | | | |
| Radiologic/Imaging assessment | X |  |  |  | X |  |  |
| Other assessments (e.g., immunology assays, pharmacokinetic) | X | X | X | X | X | X | X |
| Complete Case Report Forms (CRFs) | X | X | X | X | X | X | X |

# INTRODUCTION

## Study Rationale

Due to the lack of large-scale randomized controlled trial (RCT) studies as support for type I evidence, ACT has been used to improve the survival prognosis of patients after RUN, and there has been a lack of recommendation for type A treatment.

## Background

High-risk upper urinary urothelial carcinoma (UTUC) accounts for 5% of urothelial malignancies. 10%, with clinical characteristics such as high malignancy, rapid progress, and poor prognosis. Radical surgery (RNU) is still the main treatment for patients with UTUC. European Association of Urology (EAU) 2017 guidelines suggest that platinum-containing chemotherapy can benefit overall survival (os) and disease-free survival (DFS) for UTUC patients, National Comprehensive Cancer Network (NCCN) 2017 The guidelines recommend that patients with stage T2 and above and those who have developed lymphatic metastasis should receive adjuvant chemotherapy (ACT). However, due to the lack of large-scale randomized controlled trial (RCT) studies as support for type I evidence, ACT has been used to improve the survival prognosis of patients after RUN, and there has been a lack of recommendation for type A treatment.

## Risk/Benefit Assessment

### Known Potential Risks

Surgery risk: Cardiopulmonary function anesthesia risk; major bleeding during and after surgery; risk of abdominal organ damage and large blood vessel damage due to tumor invasion and adhesion; possibility of delayed healing caused by postoperative wound infection; risk of tumor recurrence and metastasis in the short term after surgery; The contralateral kidney cannot be fully compensated after surgery and may require dialysis; perioperative lung infections, bedsores, and thrombosis risks.

Chemotherapy risks: Including the blood system: white blood cells, granulocytes, platelets, hemoglobin decline; urinary system: urea nitrogen, creatinine increase, hematuria; digestive system: nausea, vomiting, diarrhea, constipation, elevated alanine aminotransferase, alkaline phosphatase Increased bilirubin; skin / oral mucosa: ulcers, erythema, pruritus, herpes, hair loss, phlebitis; cardiovascular system: arrhythmia, cardiac insufficiency, pericarditis.

### Known Potential Benefits

Not applicable

### Assessment of Potential Risks and Benefits

You will not benefit directly from participating in this study. Your participation will help to explore the clinical significance of improving the survival prognosis of patients with ACT after high-risk upper urinary UTUC radical surgery. If you participate in this study, we will provide you with subject health counseling or related healthcare services.

# OBJECTIVES AND ENDPOINTS

*For purposes of registration and reporting to ClinicalTrials.gov, the terms Objectives and Endpoints as used in this template align with the terms Primary Purpose and Outcome Measures in ClinicalTrials.gov, respectively. Provide a description of the study objectives and endpoints, as well as a justification for selecting the particular endpoints, in the table format included below*. *This will provide clear articulation of how the selected primary and secondary endpoint(s) are linked to achieving the primary and secondary objectives and an explanation of why endpoint(s) were chosen.* *Data points collected in the study should support an objective or have a regulatory purpose. Therefore, careful consideration should be given prospectively to the amount of data needed to support the study’s objectives.*

An objective is the purpose for performing the study in terms of the scientific question to be answered. Express each objective as a statement of purpose (e.g., to assess, to determine, to compare, to evaluate) and include the general purpose (e.g., efficacy, effectiveness, safety) and/or specific purpose (e.g., dose-response, superiority to placebo, effect of an intervention on disease incidence, disease severity, or health behavior).

*A study endpoint is a specific measurement or observation to assess the effect of the study variable (study intervention). Study endpoints should be prioritized and should correspond to the study objectives and hypotheses being tested. Give succinct, but precise definitions of the study endpoints used to address the study’s primary objective and secondary objectives (e.g., specific laboratory tests that define safety or efficacy, clinical assessments of disease status, assessments of psychological characteristics, patient reported outcomes, behaviors or health outcomes). Include the study visits or time points at which data will be recorded or samples will be obtained. Describe how endpoint(s) will be adjudicated, if applicable.*

*Primary and secondary endpoints should be adjusted for multiplicity. If a claim is sought for the secondary endpoints, the statistical plan for adjustment for multiplicity should be aligned with those objectives.*

| OBJECTIVES | ENDPOINTS | JUSTIFICATION FOR ENDPOINTS |
| --- | --- | --- |
| Primary |  |  |
| Blood routine and biochemical examination, chest and abdomen CT review, In addition, in patients with ACT, Chemotherapy Toxicity Scale. | PFS (time to radiographic progression) | 1. Imaging advances suggest disease progression |
| Secondary |  |  |
| Blood routine and biochemical examination, chest and abdomen CT review, In addition, in patients with ACT, Chemotherapy Toxicity Scale. | OS (time to death regardless of cause)  CSS (time elapsed from RNU to cancer death) | 1. Counting patient survival time |

# STUDY DESIGN

## Overall Design

To assess the oncologic outcomes of radical nephroureterectomy(RUN)combined with adjuvant chemotherapy(ACT)in patients with high risk upper tract urothelial carcinoma(UTUC)．Methods One—hundred—thirty-four individuals with high—risk UTUC who underwent RUN with or without ACT were evaluated.Cox proportional hazard model and Kaplan—Meier analysis were used to determine overall and cancer specific survival in the cohort．

## Scientific Rationale for Study Design

Comparison of the effects of radical surgery and surgery combined with chemotherapy using a parallel control method

## Justification for Dose

Citabine (Eli Lillyand Company, USA) in combination with cisplatin (Qilu Pharmaceutical Co., Ltd., China) for a 3-week chemotherapy regimen (gemcitabine 1,000 mg / m^2^ intravenous drip on days 1 and 8; cisplatin 70 mg / m^2^. Intravenous

## End of Study Definition

PFS (time to radiographic progression)

Secondary Endpoints: OS (time to death regardless of cause)

CSS (time elapsed from RNU to cancer death)

# STUDY POPULATION

## Inclusion Criteria

1. Provision of signed and dated informed consent form
2. Stated willingness to comply with all study procedures and availability for the duration of the study
3. Human criteria for high-risk UTUC patients: preoperative endoscopic biopsy or radical surgery confirmed pathologically as high-grade urothelial cancer, tumor volume greater than 1 cm, multiple lesions, previous bladder tumor resection or radical total cystectomy Surgery, meeting any of the above criteria, can be identified as high-risk UTUC patients

## Exclusion Criteria

< Low-risk UTUC patients, patients who have received preoperative neoadjuvant chemotherapy, patients who have received GC chemotherapy for bladder tumors, patients with other pathological types of tumors, patients with distant metastases, patients with interrupted follow-up, preoperative Patients with significant "kinetic" function, patients with glomerular filtration rate (eGFR) <60 ml · min, (1.73 m2), and patients with severe cardiopulmonary disease>

## Lifestyle Considerations

Not applicable

## Screen Failures

All subjects who filled out the informed consent form and screened for the randomized experiment, regardless of when and why they withdrew, did not complete the observation period prescribed by the protocol, were all cases of shedding.

When the subject withdraws, the investigator should contact the subject as much as possible by going to the door, making an appointment for follow-up, or by phone. Ask for the reason, record the last treatment time, and complete the assessment items that can be completed.

Withdrawal from the test case due to allergic reactions, adverse reactions, and ineffective treatment. Therefore, corresponding treatment measures should be taken based on the actual conditions of the subject's experiments.

For the case of shedding, the researcher will fill in the detailed reason for shedding in the case report form, and the statistical analysis should be combined with the actual situation. Try to control the shedding rate.

## Strategies for Recruitment and Retention

1.Recruitment and screening of Chinese patients attending our hospital through posters

2. Provide free medical consultations to patients and keep in touch with patients to reduce missed visits

3. You will not benefit directly from participating in this study. Your participation will help to explore the clinical significance of improving the survival prognosis of patients with ACT after high-risk upper urinary UTUC radical surgery. If you participate in this study, we will provide you with subject health counseling or related healthcare services.

# STUDY INTERVENTION

## Study Intervention(s) Administration

### Study Intervention Description

Sitabine (Eli Lillyand Company, USA) and Cisplatin (Qilu Pharmaceutical Co., Ltd., China)

### Dosing and Administration

Three-week chemotherapy regimen (Gemcitabine 1,000 mg / m2 intravenously infusion on days 1 and 8; Cisplatin 70 mg / m2 intravenously in divided doses on days 2, 3, and 4)

## Preparation/Handling/Storage/Accountability

*No text is to be entered in this section; rather it should be included under the relevant subheadings below.*

### Acquisition and accountability

Not applicable

### Formulation, Appearance, Packaging, and Labeling

Sitabine (Eli Lillyand Company, USA) and Cisplatin (Qilu Pharmaceutical Co., Ltd., China)

### Product Storage and Stability

The medicines we apply are produced by the same company and stored and transported in strict accordance with standards

### Preparation

Gemcitabine 1 000 mg / m2 was infused intravenously on days 1 and 8; cisplatin 70 mg / m2 was administered intravenously in divided doses on days 2, 3, and 4.

## Measures to Minimize Bias: Randomization and Blinding

Apply SAS system to compile random schedule according to the principle of block randomization

## Study Intervention Compliance

Notify patients of treatments by filling out participant medication diaries and calling regularly

## Concomitant Therapy

### Rescue Medicine

Application of anti-chemotoxic drugs such as：

Withdrawal of chemotherapeutics, antiemetics, application of nutritional drugs

# STUDY INTERVENTION DISCONTINUATION AND PARTICIPANT DISCONTINUATION/WITHDRAWAL

## Discontinuation of Study Intervention

[Discontinuation from <study intervention> does not mean discontinuation from the study, and remaining study procedures should be completed as indicated by the study protocol. If a clinically significant finding is identified (including, but not limited to changes from baseline) after enrollment, the investigator or qualified designee will determine if any change in participant management is needed. Any new clinically relevant finding will be reported as an adverse event (AE).

The data to be collected at the time of study intervention discontinuation will include the following:

Blood routine and biochemical examination, chest and abdomen CT review. In addition, in patients with ACT,

## Participant Discontinuation/Withdrawal from the Study

Participants are free to withdraw from participation in the study at any time upon request.

An investigator may discontinue or withdraw a participant from the study for the following reasons:

- Pregnancy
- Significant study intervention non-compliance
- If any clinical adverse event (AE), laboratory abnormality, or other medical condition or situation occurs such that continued participation in the study would not be in the best interest of the participant
- Disease progression which requires discontinuation of the study intervention
- If the participant meets an exclusion criterion (either newly developed or not previously recognized) that precludes further study participation
- Participant unable to receive <study intervention> for 4weeks

The reason for participant discontinuation or withdrawal from the study will be recorded on the <specify> Case Report Form (CRF). Subjects who sign the informed consent form and are randomized but do not receive the study intervention may be replaced. Subjects who sign the informed consent form, and are randomized and receive the study intervention, and subsequently withdraw, or are withdrawn or discontinued from the study, will be replaced.

<Insert text>

## Lost to Follow-Up

A participant will be considered lost to follow-up if he or she fails to return for scheduled visits and is unable to be contacted by the study site staff.

The following actions must be taken if a participant fails to return to the clinic for a required study visit:

- The site will attempt to contact the participant and reschedule the missed visit <specify time frame> and counsel the participant on the importance of maintaining the assigned visit schedule and ascertain if the participant wishes to and/or should continue in the study.
- Before a participant is deemed lost to follow-up, the investigator or designee will make every effort to regain contact with the participant (where possible, 3 telephone calls and, if necessary, a certified letter to the participant’s last known mailing address or local equivalent methods). These contact attempts should be documented in the participant’s medical record or study file.
- Should the participant continue to be unreachable, he or she will be considered to have withdrawn from the study with a primary reason of lost to follow-up.]

# STUDY ASSESSMENTS AND PROCEDURES

## Efficacy Assessments

Blood routine and biochemical examination, chest and abdomen CT review, In addition, in patients with ACT, Chemotherapy Toxicity Scale.

## Safety and Other Assessments

Blood routine and biochemical examination, chest and abdomen CT review, In addition, in patients with ACT, Chemotherapy Toxicity Scale.

## Adverse Events and Serious Adverse Events

### Definition of Adverse Events (AE)

Adverse event means any untoward medical occurrence associated with the use of an intervention in humans, whether or not considered intervention-related.

### Definition of Serious Adverse Events (SAE)

An adverse event (AE) or suspected adverse reaction is considered "serious" if, in the view of either the investigator or sponsor, it results in any of the following outcomes: death, a life-threatening adverse event, inpatient hospitalization or prolongation of existing hospitalization, a persistent or significant incapacity or substantial disruption of the ability to conduct normal life functions, or a congenital anomaly/birth defect. Important medical events that may not result in death, be life-threatening, or require hospitalization may be considered serious when, based upon appropriate medical judgment, they may jeopardize the participant and may require medical or surgical intervention to prevent one of the outcomes listed in this definition. Examples of such medical events include allergic bronchospasm requiring intensive treatment in an emergency room or at home, blood dyscrasias or convulsions that do not result in inpatient hospitalization, or the development of drug dependency or drug abuse.

### Classification of an Adverse Event

#### Severity of Event

For adverse events (AEs) not included in the protocol defined grading system, the following guidelines will be used to describe severity.

- **Mild** – Events require minimal or no treatment and do not interfere with the participant’s daily activities.
- **Moderate** – Events result in a low level of inconvenience or concern with the therapeutic measures. Moderate events may cause some interference with functioning.
- **Severe** – Events interrupt a participant’s usual daily activity and may require systemic drug therapy or other treatment. Severe events are usually potentially life-threatening or incapacitating. Of note, the term “severe” does not necessarily equate to “serious”.

#### Relationship to Study INTERVENTION

All adverse events (AEs) must have their relationship to study intervention assessed by the clinician who examines and evaluates the participant based on temporal relationship and his/her clinical judgment. The degree of certainty about causality will be graded using the categories below. In a clinical trial, the study product must always be suspect.

- **Definitely Related** – There is clear evidence to suggest a causal relationship, and other possible contributing factors can be ruled out. The clinical event, including an abnormal laboratory test result, occurs in a plausible time relationship to study intervention administration and cannot be explained by concurrent disease or other drugs or chemicals. The response to withdrawal of the study intervention (dechallenge) should be clinically plausible. The event must be pharmacologically or phenomenologically definitive, with use of a satisfactory rechallenge procedure if necessary.
- **Probably Related** – There is evidence to suggest a causal relationship, and the influence of other factors is unlikely. The clinical event, including an abnormal laboratory test result, occurs within a reasonable time after administration of the study intervention, is unlikely to be attributed to concurrent disease or other drugs or chemicals, and follows a clinically reasonable response on withdrawal (dechallenge). Rechallenge information is not required to fulfill this definition.
- **Potentially Related** – There is some evidence to suggest a causal relationship (e.g., the event occurred within a reasonable time after administration of the trial medication). However, other factors may have contributed to the event (e.g., the participant’s clinical condition, other concomitant events). Although an AE may rate only as “possibly related” soon after discovery, it can be flagged as requiring more information and later be upgraded to “probably related” or “definitely related”, as appropriate.
- **Unlikely to be related** – A clinical event, including an abnormal laboratory test result, whose temporal relationship to study intervention administration makes a causal relationship improbable (e.g., the event did not occur within a reasonable time after administration of the study intervention) and in which other drugs or chemicals or underlying disease provides plausible explanations (e.g., the participant’s clinical condition, other concomitant treatments).
- **Not Related** – The AE is completely independent of study intervention administration, and/or evidence exists that the event is definitely related to another etiology. There must be an alternative, definitive etiology documented by the clinician.

#### Expectedness

Luo Yong will be responsible for determining whether an adverse event (AE) is expected or unexpected. An AE will be considered unexpected if the nature, severity, or frequency of the event is not consistent with the risk information previously described for the study intervention.

### Time Period and Frequency for Event Assessment and Follow-Up

The occurrence of an adverse event (AE) or serious adverse event (SAE) may come to the attention of study personnel during study visits and interviews of a study participant presenting for medical care, or upon review by a study monitor.

All AEs including local and systemic reactions not meeting the criteria for SAEs will be captured on the appropriate case report form (CRF). Information to be collected includes event description, time of onset, clinician’s assessment of severity, relationship to study product (assessed only by those with the training and authority to make a diagnosis), and time of resolution/stabilization of the event. All AEs occurring while on study must be documented appropriately regardless of relationship. All AEs will be followed to adequate resolution.

Any medical condition that is present at the time that the participant is screened will be considered as baseline and not reported as an AE. However, if the study participant’s condition deteriorates at any time during the study, it will be recorded as an AE.

Changes in the severity of an AE will be documented to allow an assessment of the duration of the event at each level of severity to be performed. AEs characterized as intermittent require documentation of onset and duration of each episode.

Wei Dechao will record all reportable events with start dates occurring any time after informed consent is obtained until 7 (for non-serious AEs) or 30 days (for SAEs) after the last day of study participation. At each study visit, the investigator will inquire about the occurrence of AE/SAEs since the last visit. Events will be followed for outcome information until resolution or stabilization.

### Adverse Event Reporting

Wei Dechao will record all reportable events, screening for adverse events. Report to Luo Yong, the responsible person within 24 hours. And the solution is given by the expert group.

### Serious Adverse Event Reporting

The study clinician will immediately report to the sponsor any serious adverse event, whether or not considered study intervention related, including those listed in the protocol or investigator brochure and must include an assessment of whether there is a reasonable possibility that the study intervention caused the event. Study endpoints that are serious adverse events (e.g., all-cause mortality) must be reported in accordance with the protocol unless there is evidence suggesting a causal relationship between the study intervention and the event (e.g., death from anaphylaxis). In that case, the investigator must immediately report the event to the sponsor.

All serious adverse events (SAEs) will be followed until satisfactory resolution or until the site investigator deems the event to be chronic or the participant is stable. Other supporting documentation of the event may be requested by the Data Coordinating Center (DCC)/study sponsor and should be provided as soon as possible.

The study sponsor will be responsible for notifying the Food and Drug Administration (FDA) of any unexpected fatal or life-threatening suspected adverse reaction as soon as possible, but in no case later than 7 calendar days after the sponsor's initial receipt of the information. In addition, the sponsor must notify FDA and all participating investigators in an Investigational New Drug (IND) safety report of potential serious risks, from clinical trials or any other source, as soon as possible, but in no case later than 15 calendar days after the sponsor determines that the information qualifies for reporting.

### Reporting Events to Participants

Not applicable.

### Events of Special Interest

Not applicable

### Reporting of Pregnancy

Not applicable

## Unanticipated Problems

### Definition of Unanticipated Problems (UP)

The Office for Human Research Protections (OHRP) considers unanticipated problems involving risks to participants or others to include, in general, any incident, experience, or outcome that meets **all** of the following criteria:

- Unexpected in terms of nature, severity, or frequency given (a) the research procedures that are described in the protocol-related documents, such as the Institutional Review Board (IRB)-approved research protocol and informed consent document; and (b) the characteristics of the participant population being studied;
- Related or possibly related to participation in the research (“possibly related” means there is a reasonable possibility that the incident, experience, or outcome may have been caused by the procedures involved in the research);
- Suggests that the research places participants or others at a greater risk of harm (including physical, psychological, economic, or social harm) than was previously known or recognized.

### Unanticipated Problem Reporting

The investigator will report unanticipated problems (UPs) to the reviewing Institutional Review Board (IRB) and to the Data Coordinating Center (DCC)/lead principal investigator (PI). The UP report will include the following information:

- Protocol identifying information: protocol title and number, PI’s name, and the IRB project number;
- A detailed description of the event, incident, experience, or outcome;
- An explanation of the basis for determining that the event, incident, experience, or outcome represents an UP;
- A description of any changes to the protocol or other corrective actions that have been taken or are proposed in response to the UP.

To satisfy the requirement for prompt reporting, UPs will be reported using the following timeline:

- UPs that are serious adverse events (SAEs) will be reported to the IRB and to the DCC/study sponsor within <insert timeline in accordance with policy> of the investigator becoming aware of the event.
- Any other UP will be reported to the IRB and to the DCC/study sponsor within <insert timeline in accordance with policy> of the investigator becoming aware of the problem.
- All UPs should be reported to appropriate institutional officials (as required by an institution’s written reporting procedures), the supporting agency head (or designee), and the Office for Human Research Protections (OHRP) within <insert timeline in accordance with policy> of the IRB’s receipt of the report of the problem from the investigator.]

*Additional example text, applicable for device protocol:*

[An investigator shall submit to the sponsor and to the reviewing Institutional Review Board (IRB) a report of any unanticipated adverse device effect occurring during an investigation as soon as possible, but in no event later than 10 working days after the investigator first learns of the effect (21 CFR 812.150(a)(1)), A sponsor who conducts an evaluation of an unanticipated adverse device effect under 812.46(b) shall report the results of such evaluation to the Food and Drug Administration (FDA) and to all reviewing IRB's and participating investigators within 10 working days after the sponsor first receives notice of the effect. Thereafter the sponsor shall submit such additional reports concerning the effect as FDA requests (21 CFR 812.150(b)(1)).

### Reporting Unanticipated Problems to Participants

Not applicable.

# STATISTICAL CONSIDERATIONS

## Statistical Hypotheses

Primary Efficacy Endpoint(s): PFS (time to radiographic progression)

Secondary Efficacy Endpoint(s): OS (time to death regardless of cause)

CSS (time elapsed from RNU to cancer death)

## Sample Size Determination

- Test statistic: T-test for measurement data and x2 test for count data. Cox proportional hazard model was established, and Kaplan-Meier algorithm was used to analyze OS and CSS curves.
- Null and alternative hypotheses

There is no difference between radical surgery combined with adjuvant chemotherapy and radical surgery

There is a difference between radical surgery combined with adjuvant chemotherapy and radical surgery

- Type I error rate (alpha)
- Power level (e.g., 80% power)
- Assumed event rate for dichotomous outcome (or mean and variance of continuous outcome) for each study arm, justified and referenced by historical data as much as possible

Statistical method used to calculate the sample size, T-test for measurement data and x2 test for count data. Cox proportional hazard model was established, and Kaplan-Meier algorithm was used to analyze OS and CSS curves.

## Populations for Analyses

- Intention-to-Treat (ITT) Analysis Dataset (i.e., all randomized participants)
- Modified Intention-to-Treat Analysis Dataset (e.g., participants who took at least one dose of study intervention and/or have some particular amount of follow-up outcome data)
- Safety Analysis Dataset: defines the subset of participants for whom safety analyses will be conducted (e.g., participants who took at least one dose of study intervention)
- Per-Protocol Analysis Dataset: defines a subset of the participants in the full analysis (ITT) set who complied with the protocol sufficiently to ensure that these data would be likely to represent the effects of study intervention according to the underlying scientific model (e.g., participants who took at least 80% of study intervention for 80% of the days within the maintenance period)
- Other Datasets that may be used for sensitivity analyses

## Statistical Analyses

### General Approach

SPSS 20.0 software was used for statistical analysis. Measurement data was tested by radon and count data was tested by x2. Establish Cox proportional hazard model, apply Kaplan-Meier algorithm to analyze OS and CSS curves

### Analysis of the Primary Efficacy Endpoint(s)

PFS (time to radiographic progression)

### Analysis of the Secondary Endpoint(s)

OS (time to death regardless of cause)

CSS (time elapsed from RNU to cancer death)

### Safety Analyses

The drugs used in this experiment have been verified

### Baseline Descriptive Statistics

Not applicable

### Planned Interim Analyses

Not applicable

### Sub-Group Analyses

Study intervention only for use in adult

### Tabulation of Individual participant Data

Not applicable

### Exploratory Analyses

Not applicable

# SUPPORTING DOCUMENTATION AND OPERATIONAL CONSIDERATIONS

## Regulatory, Ethical, and Study Oversight Considerations

### Informed Consent Process

#### Consent/assent and Other Informational Documents Provided to participants

Prospective randomized controlled research project "Effect of radical surgery combined with adjuvant chemotherapy on survival prognosis of patients with high-risk upper urinary urothelial carcinoma" informed consent

This project has been reviewed and demonstrated by the Ethics Committee of Capital Medical University

Item Number: CMU-2010003X

Project manager:

Jiang Yongguang Chief Physician / Professor Director of Urology, Beijing Anzhen Hospital, Capital Medical University

Luo Yong Deputy Chief Physician Urology, Beijing Anzhen Hospital, Capital Medical University

Dear patient,

We are going to conduct a study: "Effects of radical surgery combined with adjuvant chemotherapy on survival prognosis of patients with high-risk upper urinary urothelial cancer". Your specific situation meets the eligibility criteria for this study. Therefore, we invite you to participate in this project. the study. This informed consent form will introduce you to the purpose, steps, benefits and risks of this research, please read it carefully before deciding whether to participate. When the researcher explains and discusses your informed consent, you can ask questions at any time and ask him / her to explain to you what is difficult to understand. You can discuss it with your family, friends, and your treating doctor before making a decision.

1. Why did this study?

High-risk upper urinary urothelial carcinoma (UTUC) accounts for 5% to 10% of urothelial malignancies. It has clinical features such as high malignancy, rapid progress, and poor prognosis. Radical surgery (RNU) is currently the main treatment for UTUC patients. means. How to further improve and improve the survival prognosis of patients with UTUC after radical surgery is a matter of great concern. At this stage, professional guidelines published by the European Urological Association (EAU) and the National Comprehensive Cancer Network (NCCN) suggest that chemotherapy with platinum drugs can improve overall survival (OS) and delay disease progression (DFS) in UTUC patients. However, this conclusion lacks large-scale randomized controlled studies as important evidence for the time being. Therefore, there is an urgent need to carry out related research work to demonstrate whether postoperative chemotherapy can indeed improve the survival prognosis of high-risk UTUC patients.

2. Who will be invited to participate in this study?

The research doctor will determine whether you are suitable to participate in this study based on your actual situation and the exclusion criteria in the research plan. The admission criteria for high-risk UTUC patients adopted in this study refer to the EAU guidelines: pathologically confirmed high-grade urothelial cancer, tumors larger than 1 cm, multiple lesions, previous bladder tumor resection or radical total cystectomy, Those who meet any of the above criteria may be considered for entry into this study. Exclusion criteria: patients who had received preoperative neoadjuvant chemotherapy, patients who had received GC chemotherapy for bladder tumors, patients with tumors of other pathological types, patients with distant metastases, patients with interrupted follow-up, preoperative "kidney kidney "Patients with significant abnormal function, glomerular filtration rate (eGFR <60 ml / min), and patients with severe cardiopulmonary disease, met any of the criteria were excluded from this study.

3. How many people will participate in this study?

This study plans to recruit 150 subjects.

4. What does this study include?

Collect and record your preoperative examination data: chest and abdomen CT, CTU, isotope bone imaging, blood routine and biochemical examination;

⑵. The first year of follow-up period: review of blood routine and biochemical examination every 3 months, and chest and abdomen CT every 6 months; from the second year of follow-up period: annual review of chest and abdomen CT and isotope bone imaging, as well as blood routine and Biochemical tests.

(3) Monitor the possible toxicity of chemotherapy patients, and classify, scale, and statistics according to the CACTE 4.0 standard.

Endpoint events: overall survival, tumor-specific survival, and disease-free survival.

5. How long will this study last?

The initial follow-up is expected to be 3 years, and the follow-up time may be extended if necessary.

6. What are the risks of participating in this study?

Surgical risk: Anesthesia risk of cardiopulmonary function; major bleeding during and after surgery; risk of abdominal organ damage and large vessel damage due to tumor invasion and adhesion; possibility of delayed healing due to postoperative wound infection; tumor recurrence within short period The risk of metastasis; the contralateral kidney cannot be fully compensated for dialysis after radical operation; the risk of pulmonary infection, bedsores, and thrombosis during perioperative period.

化疗. Chemotherapy risk: including blood system: white blood cells, granulocytes, platelets, hemoglobin decline; urinary system: urea nitrogen, creatinine elevation, hematuria; digestive system: nausea, vomiting, diarrhea, constipation, alanine aminotransferase, alkaline Elevated phosphatase and bilirubin; skin / oral mucosa: ulcers, erythema, pruritus, herpes, hair loss, phlebitis; cardiovascular system: arrhythmia, cardiac insufficiency, pericarditis.

7. What are the benefits of participating in this study?

You will not benefit directly from participating in this study. Your participation will help to explore the clinical significance of improving the survival prognosis of patients with ACT after high-risk upper urinary UTUC radical surgery. If you participate in this study, we will provide you with subject health counseling or related healthcare services.

8. Do I have to participate in and complete this study?

Whether you participate in this research is entirely voluntary. If you don't want to, you can refuse to participate, this will not have any negative impact on your current or future medical treatment. Even if you have agreed to participate, you can change your mind at any time and tell the researcher to withdraw from the study. Your withdrawal will not affect your access to normal medical services.

Note: After the subject withdraws, it is necessary to make clear that new data related to the subject will not be collected in the future, and to explain to the subject how to deal with the previously collected research data and data withdrawn due to adverse reactions. If a subject withdraws from the study partway through, the researcher should destroy the collected (involved in this study) subject information in a timely manner and not use it unless he or she no longer continues to collect his personal information. For the collected data after the subject withdraws, you can refer to it. When you decide to withdraw from the study, we will stop collecting new data related to this study. We will not continue to use or disclose the collected data. Information about the study and destroy it in a timely manner.

9. Description of costs and compensation for participating in the study

The costs involved in participating in this study need to be borne by the subject, without compensation, please know.

10． Treatment of occurrence of research-related injuries?

When your health is harmed by participating in this research, please inform the researcher (010-64456096), we will take all medical measures in time to treat it in full and pay compensation in accordance with current national laws.

11. Will my information be kept confidential?

If you decide to participate in this study, your personal information in the study and in the study is confidential. Your specimen will be identified by a study number instead of your name. Information that identifies you will not be disclosed to anyone other than a research member unless you have your permission. All research members are required to keep your identity confidential. Your files will be kept in locked file cabinets for research personnel only. To ensure that research is conducted in accordance with regulations, members of government administrations or ethics committees can access your personal data at the research unit as required. When this research is published, no personal information about you will be disclosed.

12. Who can I contact if I have questions or difficulties?

If you have any questions related to this study, please contact Dr. Feng Bingfu or Dr. Wei Dechao at 010-64456096. Note: The contact number is answered 24 hours a day.

13. Subject statement:

The researcher explained the research background, purpose, steps, risks, and benefits of (project name). I have enough time and opportunities to ask questions, and I am satisfied with the answers made by the researcher. I know who I should contact when I have a question or want more information, I agree to participate in the study and the researchers in the study use the data in my medical records.

Subject: Date:

(When the subject is lacking or insufficiently informed, add or replace the following methods)

Legal representative: Relationship with patient: Date:

Visiting doctor: Date:

Project Leader: Date:

#### Consent Procedures and Documentation

Informed consent is a process that is initiated prior to the individual’s agreeing to participate in the study and continues throughout the individual’s study participation. Consent forms will be Institutional Review Board (IRB)-approved and the participant will be asked to read and review the document. The investigator will explain the research study to the participant and answer any questions that may arise. A verbal explanation will be provided in terms suited to the participant’s comprehension of the purposes, procedures, and potential risks of the study and of their rights as research participants. Participants will have the opportunity to carefully review the written consent form and ask questions prior to signing. The participants should have the opportunity to discuss the study with their family or surrogates or think about it prior to agreeing to participate. The participant will sign the informed consent document prior to any procedures being done specifically for the study. Participants must be informed that participation is voluntary and that they may withdraw from the study at any time, without prejudice. A copy of the informed consent document will be given to the participants for their records. The informed consent process will be conducted and documented in the source document (including the date), and the form signed, before the participant undergoes any study-specific procedures. The rights and welfare of the participants will be protected by emphasizing to them that the quality of their medical care will not be adversely affected if they decline to participate in this study.

### Study Discontinuation and Closure

This study may be temporarily suspended or prematurely terminated if there is sufficient reasonable cause. Written notification, documenting the reason for study suspension or termination, will be provided by the suspending or terminating party to <study participants, investigator, funding agency, the Investigational New Drug (IND) or Investigational Device Exemption (IDE) sponsor and regulatory authorities>. If the study is prematurely terminated or suspended, the Principal Investigator (PI) will promptly inform study participants, the Institutional Review Board (IRB), and sponsor and will provide the reason(s) for the termination or suspension. Study participants will be contacted, as applicable, and be informed of changes to study visit schedule.

Circumstances that may warrant termination or suspension include, but are not limited to:

- Determination of unexpected, significant, or unacceptable risk to participants
- Demonstration of efficacy that would warrant stopping
- Insufficient compliance to protocol requirements
- Data that are not sufficiently complete and/or evaluable
- Determination that the primary endpoint has been met
- Determination of futility

Study may resume once concerns about safety, protocol compliance, and data quality are addressed, and satisfy the sponsor, IRB and/or Food and Drug Administration (FDA).

### Confidentiality and Privacy

Participant confidentiality and privacy is strictly held in trust by the participating investigators, their staff, and the sponsor(s) and their interventions. This confidentiality is extended to cover testing of biological samples and genetic tests in addition to the clinical information relating to participants. Therefore, the study protocol, documentation, data, and all other information generated will be held in strict confidence. No information concerning the study or the data will be released to any unauthorized third party without prior written approval of the sponsor.

All research activities will be conducted in as private a setting as possible.

The study monitor, other authorized representatives of the sponsor, representatives of the Institutional Review Board (IRB), regulatory agencies or pharmaceutical company supplying study product may inspect all documents and records required to be maintained by the investigator, including but not limited to, medical records (office, clinic, or hospital) and pharmacy records for the participants in this study. The clinical study site will permit access to such records.

The study participant’s contact information will be securely stored at each clinical site for internal use during the study. At the end of the study, all records will continue to be kept in a secure location for as long a period as dictated by the reviewing IRB, Institutional policies, or sponsor requirements.

Study participant research data, which is for purposes of statistical analysis and scientific reporting, will be transmitted to and stored at the Chinese clinical trial. This will not include the participant’s contact or identifying information. Rather, individual participants and their research data will be identified by a unique study identification number. The study data entry and study management systems used by clinical sites and by Chinese clinical trial research staff will be secured and password protected. At the end of the study, all study databases will be de-identified and archived at the Chinese clinical trial.

### Future Use of Stored Specimens and Data

Data collected for this study will be analyzed and stored at the Anzhen Hospital. After the study is completed, the de-identified, archived data will be transmitted to and stored at the <specify name of Data Repository>, for use by other researchers including those outside of the study. Permission to transmit data to the Anzhen Hospital will be included in the informed consent.

During the conduct of the study, an individual participant can choose to withdraw consent to have biological specimens stored for future research. However, withdrawal of consent with regard to biosample storage may not be possible after the study is completed.

When the study is completed, access to study data and/or samples will be provided through the Anzhen Hospital.

### Key Roles and Study Governance

Provide the name and contact information of the Principal Investigator and the Medical Monitor.

| **Principal Investigator** | **Medical Monitor** |
| --- | --- |
| *Luo Yong* | *Wu Chaoyang* |
| *Anzhen Hospital* | *Ethics Committee of Capital Medical University* |
| *Anzhen Road 2,* *Chaoyang District, Beijing,China* | *Fengtai District, Beijing,China* |
| *+8601064456096* | *+8601064456216* |
| *luoyonganzhen@163.com* |  |

### Safety Oversight

Safety oversight will be under the direction of a Data and Safety Monitoring Board (DSMB) composed of individuals with the appropriate expertise. Members of the DSMB should be independent from the study conduct and free of conflict of interest, or measures should be in place to minimize perceived conflict of interest. The DSMB will meet at least semiannually to assess safety and efficacy data on each arm of the study. The DMSB will operate under the rules of an approved charter that will be written and reviewed at the organizational meeting of the DSMB. At this time, each data element that the DSMB needs to assess will be clearly defined.

### Clinical Monitoring

Clinical site monitoring is conducted to ensure that the rights and well-being of trial participants are protected, that the reported trial data are accurate, complete, and verifiable, and that the conduct of the trial is in compliance with the currently approved protocol/amendment(s), with International Conference on Harmonisation Good Clinical Practice (ICH GCP), and with applicable regulatory requirement(s).

- Monitoring for this study will be performed by <insert text>.
- on-site, centralized, early, for initial assessment and training versus throughout the study, and extent 100% data verification.

### Quality Assurance and Quality Control

Each clinical site will perform internal quality management of study conduct, data and biological specimen collection, documentation and completion. An individualized quality management plan will be developed to describe a site’s quality management.

Quality control (QC) procedures will be implemented beginning with the data entry system and data QC checks that will be run on the database will be generated. Any missing data or data anomalies will be communicated to the site(s) for clarification/resolution.

Following written Standard Operating Procedures (SOPs), the monitors will verify that the clinical trial is conducted and data are generated and biological specimens are collected, documented (recorded), and reported in compliance with the protocol, International Conference on Harmonisation Good Clinical Practice (ICH GCP), and applicable regulatory requirements (e.g., Good Laboratory Practices (GLP), Good Manufacturing Practices (GMP)).

The investigational site will provide direct access to all trial related sites, source data/documents, and reports for the purpose of monitoring and auditing by the sponsor, and inspection by local and regulatory authorities.

### Data Handling and Record Keeping

#### Data Collection and Management Responsibilities

Data collection is the responsibility of the clinical trial staff at the site under the supervision of the site investigator. The investigator is responsible for ensuring the accuracy, completeness, legibility, and timeliness of the data reported.

All source documents should be completed in a neat, legible manner to ensure accurate interpretation of data.

Hardcopies of the study visit worksheets will be provided for use as source document worksheets for recording data for each participant enrolled in the study. Data recorded in the electronic case report form (eCRF) derived from source documents should be consistent with the data recorded on the source documents.

Clinical data (including adverse events (AEs), concomitant medications, and expected adverse reactions data) and clinical laboratory data will be entered into <specify name of data capture system>, a 21 CFR Part 11-compliant data capture system provided by the <specify Data Coordinating Center>. The data system includes password protection and internal quality checks, such as automatic range checks, to identify data that appear inconsistent, incomplete, or inaccurate. Clinical data will be entered directly from the source documents.]

#### Study Records Retention

Study documents should be retained for a minimum of 2 years after the last approval of a marketing application in an International Conference on Harminosation (ICH) region and until there are no pending or contemplated marketing applications in an ICH region or until at least 2 years have elapsed since the formal discontinuation of clinical development of the study intervention. These documents should be retained for a longer period, however, if required by local regulations. No records will be destroyed without the written consent of the sponsor, if applicable. It is the responsibility of the sponsor to inform the investigator when these documents no longer need to be retained.

### Protocol Deviations

A protocol deviation is any noncompliance with the clinical trial protocol, International Conference on Harmonisation Good Clinical Practice (ICH GCP), or Manual of Procedures (MOP) requirements. The noncompliance may be either on the part of the participant, the investigator, or the study site staff. As a result of deviations, corrective actions are to be developed by the site and implemented promptly.

These practices are consistent with ICH GCP:

- 4.5 Compliance with Protocol, sections 4.5.1, 4.5.2, and 4.5.3
- 5.1 Quality Assurance and Quality Control, section 5.1.1
- 5.20 Noncompliance, sections 5.20.1, and 5.20.2.

It is the responsibility of the site investigator to use continuous vigilance to identify and report deviations within <specify number> working days of identification of the protocol deviation, or within <specify number> working days of the scheduled protocol-required activity. All deviations must be addressed in study source documents, reported to <specify NIH Institute or Center (IC)> Program Official and <specify Data Coordinating Center or sponsor>. Protocol deviations must be sent to the reviewing Institutional Review Board (IRB) per their policies. The site investigator is responsible for knowing and adhering to the reviewing IRB requirements. Further details about the handling of protocol deviations will be included in the MOP.

### Publication and Data Sharing Policy

This study will be conducted in accordance with the following publication and data sharing policies and regulations:

National Institutes of Health (NIH) Public Access Policy, which ensures that the public has access to the published results of NIH funded research. It requires scientists to submit final peer-reviewed journal manuscripts that arise from NIH funds to the digital archive PubMed Central upon acceptance for publication.

This study will comply with the NIH Data Sharing Policy and Policy on the Dissemination of NIH-Funded Clinical Trial Information and the Clinical Trials Registration and Results Information Submission rule. As such, this trial will be registered at ClinicalTrials.gov, and results information from this trial will be submitted to ClinicalTrials.gov. In addition, every attempt will be made to publish results in peer-reviewed journals. Data from this study may be requested from other researchers x years after the completion of the primary endpoint by contacting <specify person or awardee institution, or name of data repository>.

In addition, this study will comply with the NIH Genomic Data Sharing Policy, which applies to all NIH-funded research that generates large-scale human or non-human genomic data, as well as the use of these data for subsequent research. Large-scale data include genome-wide association studies (GWAS), single nucleotide polymorphisms (SNP) arrays, and genome sequence, transcriptomic, epigenomic, and gene expression data.

### Conflict of Interest Policy

The independence of this study from any actual or perceived influence, such as by the pharmaceutical industry, is critical. Therefore, any actual conflict of interest of persons who have a role in the design, conduct, analysis, publication, or any aspect of this trial will be disclosed and managed. Furthermore, persons who have a perceived conflict of interest will be required to have such conflicts managed in a way that is appropriate to their participation in the design and conduct of this trial. The study leadership in conjunction with the <specify NIH Institute or Center (IC)> has established policies and procedures for all study group members to disclose all conflicts of interest and will establish a mechanism for the management of all reported dualities of interest.

## Additional Considerations

Not applicable

## Abbreviations

*The list below includes abbreviations utilized in this template. However, this list should be customized for each protocol (i.e., abbreviations not used should be removed and new abbreviations used should be added to this list).*

| AE | Adverse Event |
| --- | --- |
| ANCOVA | Analysis of Covariance |
| CFR | Code of Federal Regulations |
| CLIA | Clinical Laboratory Improvement Amendments |
| CMP | Clinical Monitoring Plan |
| COC | Certificate of Confidentiality |
| CONSORT | Consolidated Standards of Reporting Trials |
| CRF | Case Report Form |
| DCC | Data Coordinating Center |
| DHHS | Department of Health and Human Services |
| DSMB | Data Safety Monitoring Board |
| DRE | Disease-Related Event |
| EC | Ethics Committee |
| eCRF | Electronic Case Report Forms |
| FDA | Food and Drug Administration |
| FDAAA | Food and Drug Administration Amendments Act of 2007 |
| FFR | Federal Financial Report |
| GCP | Good Clinical Practice |
| GLP | Good Laboratory Practices |
| GMP | Good Manufacturing Practices |
| GWAS | Genome-Wide Association Studies |
| HIPAA | Health Insurance Portability and Accountability Act |
| IB | Investigator’s Brochure |
| ICH | International Conference on Harmonisation |
| ICMJE | International Committee of Medical Journal Editors |
| IDE | Investigational Device Exemption |
| IND | Investigational New Drug Application |
| IRB | Institutional Review Board |
| ISM | Independent Safety Monitor |
| ISO | International Organization for Standardization |
| ITT | Intention-To-Treat |
| LSMEANS | Least-squares Means |
| MedDRA | Medical Dictionary for Regulatory Activities |
| MOP | Manual of Procedures |
| MSDS | Material Safety Data Sheet |
| NCT | National Clinical Trial |
| NIH | National Institutes of Health |
| NIH IC | NIH Institute or Center |
| OHRP | Office for Human Research Protections |
| PI | Principal Investigator |
| QA | Quality Assurance |
| QC | Quality Control |
| SAE | Serious Adverse Event |
| SAP | Statistical Analysis Plan |
| SMC | Safety Monitoring Committee |
| SOA | Schedule of Activities |
| SOC | System Organ Class |
| SOP | Standard Operating Procedure |
| UP | Unanticipated Problem |
| US | United States |

## Protocol Amendment History

*The table below is intended to capture changes of IRB-approved versions of the protocol, including a description of the change and rationale. A Summary of Changes table for the current amendment is located in the Protocol Title Page.*

| **Version** | **Date** | **Description of Change** | **Brief Rationale** |
| --- | --- | --- | --- |
|  |  |  |  |
|  |  |  |  |
|  |  |  |  |
|  |  |  |  |
|  |  |  |  |
|  |  |  |  |
|  |  |  |  |
|  |  |  |  |
|  |  |  |  |
|  |  |  |  |
|  |  |  |  |
|  |  |  |  |
|  |  |  |  |
|  |  |  |  |
|  |  |  |  |
|  |  |  |  |
|  |  |  |  |
|  |  |  |  |
|  |  |  |  |
|  |  |  |  |
|  |  |  |  |
|  |  |  |  |
|  |  |  |  |
|  |  |  |  |

# REFERENCES

*Include a list of relevant literature and citations for all publications referenced in the text of the protocol. Use a consistent, standard, modern format, which might be dependent upon the required format for the anticipated journal for publication (e.g., N Engl J Med, JAMA, etc.). The preferred format is International Committee of Medical Journal Editors (ICMJE). Include citations to product information such as manufacturer’s IB, package insert, and device labeling.*

*Examples:*

- ***Journal citation*** *Veronesi U, Maisonneuve P, Decensi A. Tamoxifen: an enduring star. J Natl Cancer Inst. 2007 Feb 21;99(4):258-60.*
- ***Whole book citation*** *Belitz HD, Grosch W, Schieberle P. Food chemistry. 3^rd^ rev. ed. Burghagen MM, translator. Berlin: Springer; 2004. 1070 p.*
- ***Chapter in a book citation*** *Riffenburgh RH. Statistics in medicine. 2^nd^ ed. Amsterdam (Netherlands): Elsevier Academic Press; c2006. Chapter 24, Regression and correlation methods; p. 447-86.*
- ***Web Site citation****Complementary/Integrative Medicine [Internet]. Houston: University of Texas, M.D. Anderson Cancer Center; c2007 [cited 2007 Feb 21]. Available from: http://www.manderson.org/departments/CIMER/.*
- ***Electronic Mail citation***

*Backus, Joyce. Physician Internet search behavior: detailed study [Internet]. Message to: Karen Patrias. 2007 Mar 27 [cited 2007 Mar 28]. [2 paragraphs]*

- ***References to package insert, device labeling or investigational brochure***

*Cite date accessed, version number, and source of product information.*
