# Supplementary material for: Adjuvant chemotherapy after radical nephroureterectomy improves the survival outcome of high-risk upper tract urothelial carcinoma patients with cardiovascular comorbidity
Source: Sci Rep. 2020 Oct 19;10:17674. doi: 10.1038/s41598-020-74940-x (PMC7572393; doi:10.1038/s41598-020-74940-x)
Supplement: Supplementary file 3 — Supplementary Information 3. [file 41598_2020_74940_MOESM3_ESM.doc]

**Adjuvant chemotherapy after radical nephroureterectomy improves the survival outcome of high-risk upper tract urothelial carcinoma patients with cardiovascular comorbidity**

LUO Yong*, FENG Bingfu, WEI Dechao, HAN Yili, LI Mingchuan, ZHAO Jiahui, LIN Yunhua, HOU Zhu, JIANG Yongguang

Department of Urology, Beijing Anzhen Hospital, Capital Medical University. Anzhenli Street, Chaoyang District, Beijing, 100029, PR. China.

**Peri-operative CVE Rsik Standard (ANZHEN)**

| **Low-Risk** | **Intermediate-Rlsk** | **High-Risk** | **Very High-Risk** |
| --- | --- | --- | --- |
|
| **Single Vessel Disease** | **Single Vessel Disease** | **Single Vessel Disease** | **Single Vessel Disease** |
| Vascular stenosis＜50%;  Stenosis of stent or bridge＜50%； | Vascular stenosis 50~90%;  Stenosis of stent or bridge 50~90%; | Vascular stenosis≥90%;  Stenosis of stent or bridge≥90%; | Complete occlusion of vessel, stent or bridge; |
| **Two Vessel Disease** | **Two Vessel Disease** | **Two Vessel Disease** | **Two Vessel Disease** |
| The stenosis of both vessels ＜30%; | The stenosis of both vessels no more than 50%; | The stenosis of any vessels 50~90%; | The stenosis of any vessels ≥90%;  The stenosis of both vessels 50~90%; |
| When stent or bridge open, the stenosis of another vessel ＜50%; | When stent or bridge open, the stenosis of another vessel 50~90%; | When stent or bridge open, the stenosis of another vessel ≥90%; | When stent or bridge open, another vessel complete occlusion; |
|  | When the stenosis of stent or bridge＜30%, the stenosis of another vessel＜50%; | When the stenosis of stent or bridge＜50%, the stenosis of another vessel＜90%; | The stenosis of stent or bridge≥50%;  The stenosis of another vessel≥90%; |
| **Triple Vessel Disease** | **Triple Vessel Disease** | **Triple Vessel Disease** | **Triple Vessel Disease** |
|  | The stenosis of all triple vessels＜50%; | The stenosis of two vessels ≥50%; | The stenosis of any vessel≥90%;  The stenosis of all triple vessels≥50%; |
| **Valvular Disease** | **Valvular Disease** | **Valvular Disease** | **Valvular Disease** |
| Any valve reaches mild regurgitation/stenosis; | Any valve reaches moderate regurgitation/stenosis; | Any valve reaches severe regurgitation/stenosis;  Two valves reach moderate regurgitation/stenosis; | Two or more valves reach severe regurgitation/stenosis;  Three valves reach moderate regurgitation/stenosis; |
| Replaced valve works well; | Replaced valve reaches mild regurgitation/stenosis;  Other valve reaches mild regurgitation/stenosis, when replaced valve works well; | Replaced valve reaches moderate regurgitation/stenosis;  Other valve reaches moderate regurgitation/stenosis, when replaced valve works well; | Replaced valve reaches severe regurgitation/stenosis;  Other valve reaches severe regurgitation/stenosis, when replaced valve works well;  Perivalvular leakage;  Valve excrescence; |
| **Other Conditions** | **Other Conditions** | **Other Conditions** | **Other Conditions** |
|  | The diameter of thoracic/abdominal aortic aneurysm ＜3cm; | The diameter of thoracic/abdominal aortic aneurysm 3~5cm;  Ejection fraction 30~55%;  Myocardial ischemic area of myocardial infarction patients less than 30% by myocardial radionuclide examination;  Cardiac function reaches level 2; | The diameter of thoracic/abdominal aortic aneurysm 3~5cm;  ≥5cm；  Ascending aortic aneurysm;  Ejection fraction＜30%;  Myocardial ischemic area of myocardial infarction patients more than 30% by myocardial radionuclide examination;  Ventricular aneurysm;  Combined with more than two different types of heart disease;  Cardiac function above level 3; |
